# Transmembrane Protein TMEM230, a Target of Glioblastoma Therapy

**DOI:** 10.3389/fncel.2021.703431

**Published:** 2021-11-17

**Authors:** Cinzia Cocola, Valerio Magnaghi, Edoardo Abeni, Paride Pelucchi, Valentina Martino, Laura Vilardo, Eleonora Piscitelli, Arianna Consiglio, Giorgio Grillo, Ettore Mosca, Roberta Gualtierotti, Daniela Mazzaccaro, Gina La Sala, Chiara Di Pietro, Mira Palizban, Sabino Liuni, Giuseppina DePedro, Stefano Morara, Giovanni Nano, James Kehler, Burkhard Greve, Alessio Noghero, Daniela Marazziti, Federico Bussolino, Gianfranco Bellipanni, Igea D’Agnano, Martin Götte, Ileana Zucchi, Rolland Reinbold

**Affiliations:** ^1^Institute for Biomedical Technologies, National Research Council, Milan, Italy; ^2^Consorzio Italbiotec, Milan, Italy; ^3^Department of Pharmacological and Biomolecular Sciences, University of Milan, Milan, Italy; ^4^Department of Pathophysiology and Transplantation, Università degli Studi di Milano, Fondazione IRCCS Ca’ Granda Ospedale Maggiore Policlinico, Milan, Italy; ^5^Operative Unit of Vascular Surgery, IRCCS Policlinico San Donato, San Donato Milanese, Italy; ^6^Institute of Biochemistry and Cell Biology, Italian National Research Council, Rome, Italy; ^7^Department of Gynecology and Obstetrics, University Hospital of Münster, Münster, Germany; ^8^Department of Molecular and Translational Medicine, University of Brescia, Brescia, Italy; ^9^Institute of Neuroscience, Milan, Italy; ^10^Department of Biomedical Sciences for Health, Università degli Studi di Milano, Milan, Italy; ^11^National Institutes of Health, NIDDK, Laboratory of Cell and Molecular Biology, Bethesda, MD, United States; ^12^Department of Radiation Therapy and Radiation Oncology, University Hospital of Münster, Münster, Germany; ^13^Lovelace Biomedical Research Institute, Albuquerque, NM, United States; ^14^Department of Oncology, University of Turin, Orbassano, Italy; ^15^Laboratory of Vascular Oncology Candiolo Cancer Institute – IRCCS, Candiolo, Italy; ^16^Department of Biology, Center for Biotechnology, Sbarro Institute for Cancer Research and Molecular Medicine, Temple University, Philadelphia, PA, United States

**Keywords:** cargo vesicle transport, angiogenesis and normalization of vascular network, tumor cell migration and adhesion, anticancer and antiangiogenic therapy, glioma, kinesin motor proteins

## Abstract

Glioblastomas (GBM) are the most aggressive tumors originating in the brain. Histopathologic features include circuitous, disorganized, and highly permeable blood vessels with intermittent blood flow. These features contribute to the inability to direct therapeutic agents to tumor cells. Known targets for anti-angiogenic therapies provide minimal or no effect in overall survival of 12–15 months following diagnosis. Identification of novel targets therefore remains an important goal for effective treatment of highly vascularized tumors such as GBM. We previously demonstrated in zebrafish that a balanced level of expression of the transmembrane protein TMEM230/C20ORF30 was required to maintain normal blood vessel structural integrity and promote proper vessel network formation. To investigate whether TMEM230 has a role in the pathogenesis of GBM, we analyzed its prognostic value in patient tumor gene expression datasets and performed cell functional analysis. TMEM230 was found necessary for growth of U87-MG cells, a model of human GBM. Downregulation of TMEM230 resulted in loss of U87 migration, substratum adhesion, and re-passaging capacity. Conditioned media from U87 expressing endogenous TMEM230 induced sprouting and tubule-like structure formation of HUVECs. Moreover, TMEM230 promoted vascular mimicry-like behavior of U87 cells. Gene expression analysis of 702 patients identified that TMEM230 expression levels distinguished high from low grade gliomas. Transcriptomic analysis of patients with gliomas revealed molecular pathways consistent with properties observed in U87 cell assays. Within low grade gliomas, elevated TMEM230 expression levels correlated with reduced overall survival independent from tumor subtype. Highest level of TMEM230 correlated with glioblastoma and ATP-dependent microtubule kinesin motor activity, providing a direction for future therapeutic intervention. Our studies support that TMEM230 has both glial tumor and endothelial cell intracellular and extracellular functions. Elevated levels of TMEM230 promote glial tumor cell migration, extracellular scaffold remodeling, and hypervascularization and abnormal formation of blood vessels. Downregulation of TMEM230 expression may inhibit both low grade glioma and glioblastoma tumor progression and promote normalization of abnormally formed blood vessels. TMEM230 therefore is both a promising anticancer and antiangiogenic therapeutic target for inhibiting GBM tumor cells and tumor-driven angiogenesis.

## Introduction

Glioblastoma (GBM) is the most malignant of brain tumors, representing 15% of all tumors within the brain. Glioblastoma is characterized by extensive vascularization, invasion and tissue remodeling with few patients surviving beyond 2 years (Ushio, 1991; Puzzilli et al., 1998; Karpati et al., 1999; Ohgaki and Kleihues, 2005; Brandes et al., 2008; Birk et al., 2017; Polivka Jr., Polivka et al., 2017; Gusyatiner and Hegi, 2018; Kang et al., 2020; Widodo et al., 2021). These pathological features contribute to GBM being highly untreatable and associated with the tumor recurring following therapeutic intervention. Studies suggest that while blood vessels of the tumor microenvironment are supportive to tumor cells, existing and newly generated tumor vasculature are often permeable, making targetability of antitumor agents ineffective (Visted et al., 2003; Tate and Aghi, 2009; Thomas et al., 2014; Trevisan et al., 2014; Vartanian et al., 2014; Weathers and De Groot, 2014, 2015; Wang et al., 2016; Wirsching et al., 2016; Touat et al., 2017; Zhou et al., 2020). Anti-angiogenic therapies while effective in certain types of tumors, have proven ineffective to normalize existing and newly generated tumor vasculature or block angiogenesis for treatment in GBM patients. Why antiangiogenic treatments are not efficacious in the highly vascularized GBM remains unknown (Jain, 2013; Arrillaga-Romany and Norden, 2014; Bartolotti et al., 2014; Redzic et al., 2014; Aldape et al., 2015; Masui et al., 2016; Montano et al., 2016; Ludwig and Kornblum, 2017; Jo and Wen, 2018; Jovcevska, 2018; Gately et al., 2019; Uddin et al., 2020; Jones et al., 2021; Lopes Abath Neto and Aldape, 2021). No specific mutations and chromatin lesions linked with GBM explain the ineffectiveness of anti-angiogenic strategies. Therefore, epigenomic components of the GBM tumor environment and interactions of different cell types likely contribute to the lack of effectiveness of current therapies (Nagarajan and Costello, 2009; Kondo et al., 2014; Romani et al., 2018). The model of tumor associated angiogenic switch supports that tumor cells remodel extracellular matrix tension or secrete factors or vesicles that promote vascularization of the tumor environment (Sogno et al., 2009; Xu et al., 2016; Groblewska et al., 2020). Of particular interest is whether tumor cells with angiogenic potential or tumor associated endothelial cells induced to have aberrant angiogenic features share genes in common that have pleiotropic like properties. Recently, we identified the transmembrane protein, TMEM230 as a novel regulator of normal development associated angiogenesis in zebrafish (Carra et al., 2018). Modulation of TMEM230 expression was sufficient to affect the activities of components of the VEGF and Delta/Notch signaling pathways and induce new blood vessel formation and structural remodeling of existing blood vessels *in vivo* (Carra et al., 2018). This suggested that TMEM230 promotes aspects of angiogenesis in parallel or independently of the Delta/Notch and VEGF signaling pathways. TMEM230 has the properties of being a novel master regulator in angiogenesis. Depending on the expression level, TMEM230 could induce or recover aberrant number of endothelial cells and inhibit or promote the normal function and structural properties of blood vessels. Additionally, TMEM230 could recuperate normal function and 3D structural properties of aberrantly formed blood vessels such as induced in disease or cancer development. Therefore, precise regulated levels of TMEM230 expression may determine its role in normal or disease associated angiogenesis. Search of published and open access research on microarray, sequencing and proteomic expression analysis did not uncover any datasets to ascertain at a preliminary level whether TMEM230 was differentially expressed specifically between non-malignant glial cells and glial cells from low- or high-grade gliomas. As *TMEM230* sequence is conserved in human and zebrafish, we investigated whether *TMEM230* is expressed in human tumors and may represent a promising novel drug target for antiangiogenic or antitumor therapy to restrict GBM tumor cell properties and tumor cell promoted angiogenesis.

In this study we demonstrated that TMEM230 represents a novel pleiotropic acting gene with both intracellular and extracellular tumor and vascularization in the form of vascular mimicry and angiogenic promoting capacities. Conditioned media from U87 cells expressing TMEM230 promoted human endothelial cells to sprout and form tubule-like structures. Intracellular expression of TMEM230 in U87-MG promoted cells to migrate and organize into endothelial vessel-like structures, a process described as vascular mimicry as vascular mimicry (VM). The vascular mimicry was inhibited in U87 cells when TMEM230 was down regulated. Our new study supported that the TMEM230 expressing U87 cells may secrete extracellular components and modulate their tumor microenvironment to promote tumor cell induced VM or endothelial associated angiogenesis. Our previous research supports that proper levels of TMEM230 are necessary for normal endothelial cell sprouting and maintaining the structural integrity of blood vessels. Here we propose that aberrantly elevated levels of TMEM230 promote abnormal vascularization by driving endothelial cells to generate an abundance of defective blood vessels or glial tumor cells to form vessel like structures through VM. Moreover, TMEM230 drives glial tumor cells to promote abnormal tissue and existing blood vessel remodeling. TMEM230 therefore functions in two different cell types (glial and endothelial) to promote in tumor formation, tissue destruction and hypervascularization, and destabilization of existing normal blood vessels. Judicious and precise use of levels of TMEM230 for therapy may inhibit these tumor properties and also help to normalize blood vessel function in GBM. Human TMEM230 emerges as a promising novel target for antiangiogenic and antitumor therapies due to its pleiotropic like regulatory role in tumor associated angiogenesis and invasive and vascular mimicry properties in U87 tumor cells.

## Materials and Methods

### Patient Data Collection

mRNAseq datasets of GBM and LGG brain tumors and corresponding patient clinical data were obtained from The Cancer Genome Atlas (TCGA) (Cancer Genome Atlas Research Network, Weinstein et al., 2013), analyzed using R package TCGA2STAT (Wan et al., 2016), and normalized with RSEM (Li and Dewey, 2011). GBM and LGG datasets used for TMEM230 expression included 172 brain samples from patients with high grade (G3, G4) GlioBlastoma Multiforme (GBM) and 530 brain samples from patients with low grade gliomas that include 198 oligodendroglioma, 134 oligoastrocytoma, 197 astrocytoma. Grades 3 and 4 tumors were defined according to the American Joint Committee on Cancer AJCC. For gene expression analysis we excluded the samples of unknown grading.

### Patient RNA-Seq Gene Expression Analysis

Differential gene expression analysis was performed using DESEQ2 with a *p*-Value cut-off <0.0001 and an absolute log2 fold change cut-off >0.58 (Love et al., 2014). Functional enrichment analysis was performed using DAVID (6.8) (Huang da et al., 2009). Only terms with a corrected *p*-Value (Benjamini) < 0.05 were considered. The expression data related to the TCGA repository of LGG samples were downloaded using the TCGA2STAT R package (2) and gene expression analysis was performed using the DESEQ2 R package (version 1.30.1^1^).

### Correlation and Heatmap Analysis of TMEM230 Expression From Patient Data

Correlation of TMEM230 gene expression levels on overall survival (OS) in astrocytoma, oligoastrocytoma and oligodendroglioma patients was performed using the Kaplan-Meier plotter online tool, GRAPHPAD PRISM. For each glioma subtype, analytical groups were generated based on the median of the RSEM normalized gene expression of TMEM230. The difference in survival between groups was calculated using the software R unpaired *t*-test. The Heatmap on the 100 most variable expressed genes was generated using the R “pheatmap” package. The functional enrichment analysis of the differential expressed genes was performed with DAVID 6.8.

### RNA Isolation and RT PCR

RNA was isolated from U87 cells in which TMEM230 was down regulated or control cells using TRIzol Reagent (Thermo Fisher Scientific, 15596026) and reverse transcribed using High-Capacity cDNA Reverse Transcription Kit (Thermo Fisher Scientific, 4368813) following manufacturer’s instructions. Quantitative real-time PCR (qRT-PCR) was performed with a 7500 Real-Time PCR System (Applied Biosystem, 4345241) in total volumes of 20 uL per reaction with TMEM230 specific primers (TMEM230_FW: 5′-GATTGGCGCCTTTCTCATTATT-3′ and TMEM230_RV: 5′- CTGCCCCCCCTTTGCT-3′) and HPRT1 primers as endogenous control (HPRT1_FW: 5′-TTTGCTGACCTGCTGGATTACA-3′ and HPRT1_RV: 5′-GGTCATTACAATAGCTCTTCAGTCTGAT-3′) using the SYBR Select Master Mix (Thermo Fisher Scientific, 4472918). All reactions were performed in triplicates 3 times. ΔΔCt analysis was used to determine the relative gene expression levels after normalization with the housekeeping gene HPRT1.

### Generation and Cloning of the Endogenous TMEM230 Variant 2 (ISOFORM 2) Transcript

The TMEM230 coding sequence was amplified from cDNA obtained from U87 cDNA using primers T230infFw: 5′-gagctagcgaattcgaaTGTTATGATGCCGTCCCGTA-3′ T230infRv and 5′-atccgatttaaattcgaaCTATGGGGTGGGTGCTA-3′. Capital letters represent the nucleotide sequence that anneal with the endogenous TMEM230 transcript. Small letters are the docking sequences of the vector with the restriction sites underlined. The destination plasmid pCDH-CMV-MCS-EF1-copGFP (SBI CD511B-1) was linearized using the *Bst*BI restriction enzyme. Plasmid insert cloning was completed using In-fusion Cloning Plus (Clontech TAKARA 638920) following manufacturer’s instructions. The U87 cDNA sequence was compared to the wild-type sequence on non-malignant human patient cells to confirm that U87 cells do not contain a mutated or aberrant sequence of TMEM230.

### Cloning of Lentiviral System-Based Construct for Inhibiting TMEM230 Protein Expression

The shTMEM230 sequence (for down regulation of endogenous TMEM230) was cloned into pcDNA™6.2-GW/EmGFP using the BLOCK-iT™ Pol II miR RNAi Expression Vector Kit with EmGFP (Thermo Fisher Scientific K493600) following the manufacturer’s instruction. The following sequences were annealed to generate double stranded oligonucleotides:

**TOP**:5′-TGCTGTGTAGGTTCACTTAACATCTTgttttggccact gactgacAAGATGTTGTGAACCTACA-3′ and

**BOTTOM**:5′-cctgTGTAGGTTCACAACATCTTgtcagtcagtgg ccaaaacAAGATGTTAAGTGAACCTACAC-3′. Capital letters represent the sense and anti-sense sequences of the small hairpin RNA to be expressed for targeting the endogenous TMEM230 transcript. Small letters are the sequence forming the loop of the hairpin structure. The expression cassette of the resulting plasmid and the control vector provided in the kit (pcDNA™6.2-GW/EmGFP-miR-neg Control) were amplified by PCR using the following primers: FW 5′-GGCATGGACGAGCTGTACAA-3′ and RVNotI 5′-GTGCGGCCGCATCTGGGCCATTT-3′ (which added a *Not*I restriction site). The PCR product was cloned into the destination lentiviral vector pCDH-CMV-MCS-EF1-copGFP (SBI CD511B1), between *Bam*HI and *Not*I restriction sites.

### Lentivirus Production

Lentiviral particles were produced in HEK293T cells by transfecting pCDH or pLENTI vectors together with psPAX2 and pMD2.G (gift from Didier Trono, Addgene plasmids #12260 and #12259) as helper vectors for 2nd generation viral packaging (with a ratio 4:3:1, respectively) using the Lipofectamine™ 2000 Transfection Reagent (Thermo Fisher Scientific 11668027) following manufacturer’s instructions. Cell culture supernatants containing the lentiviral particles were harvested after 48 and 72 h, concentrated by ultracentrifugation at 120,000 rcf for 3 h and stored at –80°C for later use.

### Adherent Cell Cultures

The human brain glioblastoma U87-MG cell line was recently obtained from the ATTC and maintained in DMEM (Euroclone, ECB7501L) supplemented with 10% fetal bovine serum (FBS, Sigma, F7524), 1% Glutamine (Cambrex, BE17-605E) and 1% penicillin/streptomycin (Life Technology, 15140-122) in a humidified atmosphere of 5% CO_2_ at 37°C. Cells were cultured to an 80% level of confluence. Transduction was performed on adherent cells using lentiviral vectors (shSCR-GFP, used as control and shTMEM230-GFP for downregulating endogenous TMEM230). Cells were allowed to recover for 1 day following transduction in transduction culture medium and then culture was continued with DMEM supplemented with 10% FBS or 10% KnockOut Serum Replacement (SR, Life Technologies, 10828-028), 1% Glutamine and 1% penicillin/streptomycin, depending on the assay to be performed. Human umbilical vein endothelial cells (HUVECs) were grown in EGM2 medium consisting of Ham’s F12/DMEM-Glutamax (Life Technologies, 21765-029:31966-021) at a ratio of 1:1 supplemented with additional factors [Eurogene: (CC-4176), heparin (CC-4396A), hydrocortisone (CC-4112A), epidermal growth factor (CC-4317A), human basic fibroblast growth factor (CC-4113A), vascular endothelial growth factor (CC-4114A), ascorbic acid (CC-4116A), FBS (CC-4101A), gentamicin (CC-4381A), and R^3^ Insulin-like growth factor (R^3^IGF1, CC-4115A)]. Human umbilical vein endothelial cell were cultured in a humidified atmosphere of 5% CO_2_ at 37°C to an 80% level of confluence and medium replaced twice a week.

### Conditioned Medium Collection From U87-MG Grown With Fetal Bovine Serum or Serum Replacement Containing Medium

Conditioned media was collected from cells transduced with shSCR or shTMEM230 viral constructs cultured with 10% FBS or 10% SR, centrifuged at 2,000 × *g* for 5 min to clear supernatant of cells and cell debris and stored at –20°C for using in co-culture experiments.

### U87-MG Tubulogenesis Assay

20,000 shSCR or shTMEM230 lentivirus transduced U87-MG cells were cultured in growth factor reduced Matrigel (BD Biosciences, 356231) in 48-well plates (Greiner, Twin-Helix, 677180) using U87 (tumor) or HUVEC (vascular) medium as described in the text. Structure formation was monitored for 24, 48, and 96 h by fluorescence microscope.

### Human Umbilical Vein Endothelial Cell Angiogenesis Assays

For tubulogenesis assay, 20,000 HUVEC were plated in growth factor reduced Matrigel (BD Biosciences, 356231) in 48-well plates (Greiner, Twin-Helix, 677180) for 24 h. Tubuli forming media used were conditioned media obtained from U87shSCR cultured with FBS; U87shTMEM230 cultured with FBS; U87shSCR cultured with SR, or U87shTMEM230 cultured with SR.

For spheroid outgrowth assay, 16,000 HUVEC were suspended in 100 μl of 20% HUVEC medium containing 2% methylcellulose solution (Sigma, M7027) in 96 well plates. Spheroids were collected the day after and embedded in 60% methylcellulose containing 40% FBS and Collagen R (SERVA, Euroclone, SE4725401) at a ratio 1:1 and then layered onto a solidified bed of rat collagen in 96-well plates. After the methylcellulose-collagen mixture solidified, 100 μl of medium with or without angiogenic promoting factors were added. Angiogenetic factors used were 300 ng/ml Angiopoietin I (Sigma, Merck, SRP300) and 30 ng/ml VEGF (Sigma, Merck, V7259). Spheroids formed within 24 h from control cells with angiogenic factors. Five distinct culture conditions were examined: EGM2 (HUVEC medium) + or − angiogenic factors; condition media from U87shSCR grown in FBS + or − angiogenicfactors; U87shTMEM230 in FBS + or − angiogenic factors; U87shSCR in SR + or − angiogenic factors; and U87shTMEM230 in SR + or − angiogenic factors. Spheroids from all experimental conditions were compared to spheroids generated from control cells cultured in fresh EGM2. Control spheroids contained of approximately 800 cells within 24 h.

### Adherent Co-cultures of Human Umbilical Vein Endothelial Cells and U87-MG Cells

Drops of 50 μl containing 20,000 HUVEC were plated with shSCR or shTMEM230 transduced U87 cells at two opposite ends of a well in a 12 well-plate. U87 cells were distinguished from HUVEC because of their green fluorescence. Following cell attachment at 37°C and 5% CO2, culture media was added to immerse the adherent cells. A combination of U87 + EGM2 media at a ratio of 1:1 was used. Cell migration capacities of U87 and HUVEC were monitored for more than 10 days. Half of media was replaced with fresh media every 3 days.

### Western Blotting Analysis

For TMEM230 expression analysis proteins were prepared from conditioned media precipitated with Trichloroacetic acid (TCA, Sigma, T0699) followed by wash of methanol (412532, Carlo Erba) or U87 cells lysed on ice using Laemmli buffer (100 mM TRIS pH 7, 200 mM DTT, 20% glycerol, 4% SDS). About 10 μg and 100 μg of total protein for each sample derived from cells or conditioned media were mixed with a 6x loading dye buffer (0.375M Tris pH 6.8, 12% SDS, 60% glycerol, 0.6M DTT, 0.06% bromophenol blue) and loaded onto 10% SDS denaturing poly-acrylamide gels. After transferring proteins to a PVDF membrane (GE-Biotechnology, Euroclone, 10600021), the membrane was stained with 0.1% Ponceau S (Sigma, P3504) in 5% acetic acid (Thermo Fisher Scientific, A/0400/PB17), washed, blocked with 5% fat-dried milk (Euroclone, EMR180001) and incubated with primary antibodies. Antibodies used were polyclonal rabbit anit-C20ORF30 (TMEM230, 1:1,000, Santa Cruz, sc-85410), monoclonal mouse anti-MOESIN (1:200, Santa Cruz, sc-58806), polyclonal rabbit anti-CD138 (Syndecan-1, 1:250, Thermo Fisher Scientific, 36-2900), anti-HCAM (CD44, 1:1,000, Santa Cruz, sc-9960) and polyclonal goat anit-β-ACTIN (1:2,000, Santa Cruz, sc-1615, used as endogenous control). Donkey anti-rabbit (1:20,000, Amersham, NA934V), sheep anti-mouse (1:10,000, Amersham, NA931V), and donkey anti-goat (1:15,000, Santa Cruz, sc-2020) were used as secondary antibodies.

### Coomassie Staining

Electrophoresis gels were fixed for 30 mins in fixing solution (7% glacial acetic acid in 40% methanol) and placed in a staining solution of 0.1% Coomassie Brilliant Blue (Sigma, B0149), 7% glacial acetic acid in 40% methanol for 1 h. Gels were washed in a de-staining solution (10% acetic acid in 15% methanol), rinsed and de-stained with 25% methanol for 24 h.

### Immunofluorescence Analysis

U87 control and shTMEM230 cells were fixed with 4% Paraformaldehyde (Sigma) in 1× PBS for 10 min at room temperature (RT). Cells were incubated with a blocking buffer of 5% normal goat serum in 1× PBS. The primary antibodies used were anti-FIBRONECTIN (1:200 dilution, Chemicon International, AB2033), anti-CAVEOLIN-1 (1:100 dilution, Santa Cruz, sc-7875) and anti-phalloidin-TRITC conjugated (1:2,000, Sigma, P1951), all incubated for 2 hours at RT. Both were incubated for 2 h at RT. The cells were washed with 1× PBS and incubated with a secondary antibody of goat anti-rabbit Alexafluor-555 (1:500 dilution, A21429, Life Technologies) for 1 h at RT. Nuclei were visualized with 4′,6′-diamidino-2-phenylindole (DAPI) staining.

## Results

### Analysis of *TMEM230* Gene Expression in Human Glioma Tumors From the Cancer Genome Atlas Datasets Revealed That Human Glioblastoma Multiforme Expresses Higher Levels of *TMEM230* Compared to Lower Grade Gliomas

**FIGURE 1:**
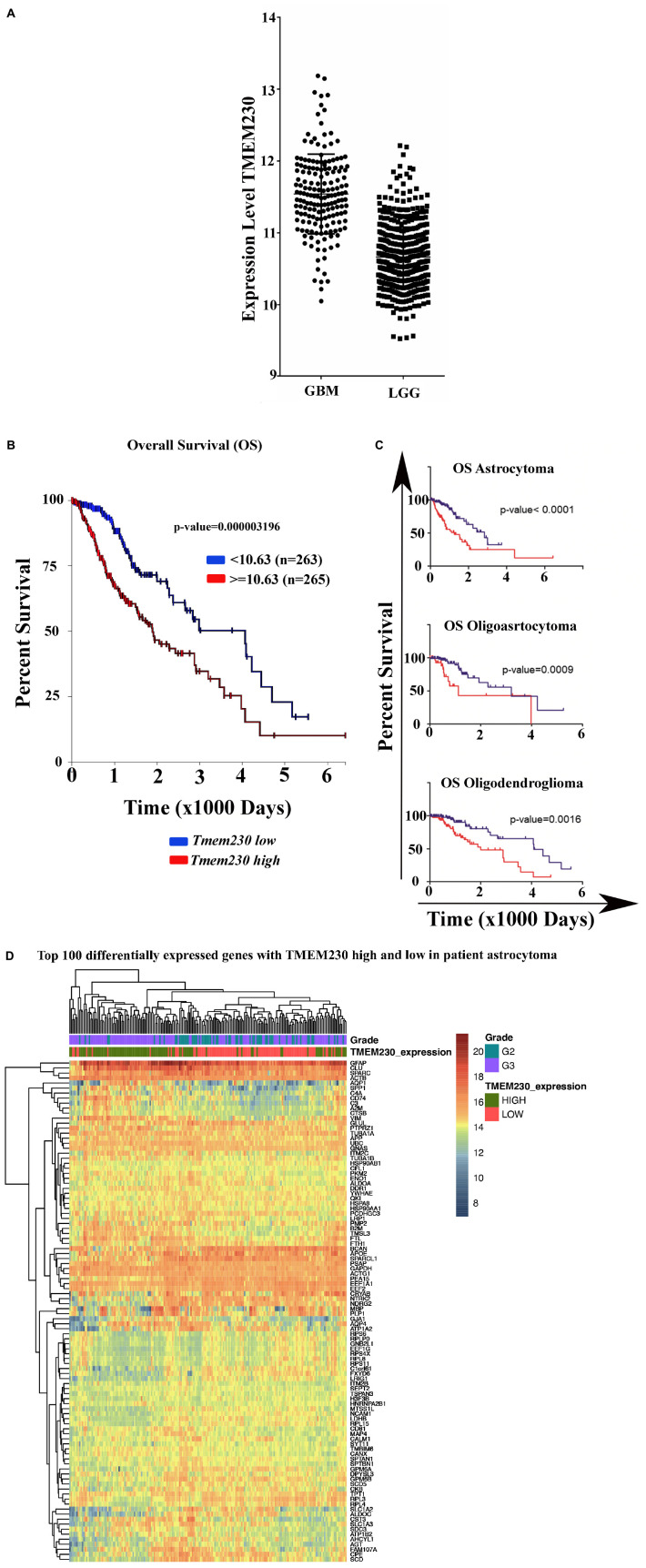
Expression of *TMEM230* in brain Glioblastoma Multiforme (GBM) and Low-Grade Gliomas (LGG) analyzed from The Cancer Genome Atlas. **(A)** Glioblastoma multiforme tumors showed significantly elevated level of *TMEM230* mRNA compared to low-grade gliomas (unpaired *t*-test *p* < 0.0001). Low-grade gliomas consist of astrocytoma, oligoastrocytoma and oligodendroglioma patient samples. **(B)** Poor prognosis was correlated with high *TMEM230 in* low-grade gliomas. **(C)** Poor prognosis was correlated with high *TMEM230* in astrocytoma (top), oligoastrocytoma (middle) and oligodendroglioma (bottom). Relationship between *TMEM230* expression levels and prognosis of low-grade gliomas affected patients indicated that lower expression of *TMEM230* was associated with increased overall survival. Each glioma subtype is indicated by the median of gene expression of *TMEM230*. B and C analyses were generated from the Kaplan-Meier test based on the expression of medium *TMEM230.* **(D)** Representative heatmap displaying the most variable expressed genes for astrocytoma using the R with “pheatmap” package for which functional enrichment was generated with DAVID program.

We recently showed that TMEM230, a transmembrane protein conserved in vertebrates and highly expressed in the vascular compartments, modulates endothelial cell sprouting and migration in Zebrafish early development (Carra et al., 2018). As the formation of new blood vessels is necessary for tumor expansion, the expression level of *TMEM230* was evaluated in several types of human glial tumors to establish whether *TMEM230* expression discriminates glioblastoma multiforme (GBM) from lower grade glial (LGG) tumors. A cohort of 530 patient samples with low grade gliomas (LGG) and a cohort of 172 patient samples with GBM from The Cancer Genome Atlas (TCGA) RNA sequencing (RNAseq) database were analyzed for *TMEM230* expression level (The Cancer Genome Atlas Research Network^2^) (Cancer Genome Atlas Research Network, Weinstein et al., 2013). *TMEM230* was significantly higher in GBM compared with brain LGG with a *P*-value ^***^P < 0.0001 using the unpaired *t*-test (Figure 1A).

### TMEM230 Low Expression Is Associated With Improved Overall Survival Rate

To investigate whether a correlation existed between *TMEM230* expression and overall survival rate, RNAseq expression datasets and clinical data of glial tumor affected patients were analyzed. The patient clinical features are summarized in Supplementary Table 1 and include gender, age, tumor size, and *TMEM230* transcript expression level.

The cohort of 530 LGG patient samples was classified according to each tumor type and the expression level of *TMEM230*. RNA sequencing data and clinical data of 198 oligodendroglioma, 134 oligoastrocytoma, 197 astrocytoma, were analyzed using the TCGA2STAT R package (Wan et al., 2016). We then investigated whether *TMEM230* expression correlated with patient prognosis using Kaplan-Meier survival analysis that determined a relationship between lower expression of *TMEM230* and increased overall survival of patients in all types of LGG (Figure 1B). Additionally, we evaluated whether *TMEM230* expression correlated the specific glioma tumor subtypes, astrocytoma (top), oligoastrocytoma (middle) and oligodendroglioma (bottom). Higher *TMEM230* was associated with worse prognosis (Figure 1C). Moreover, it was observed that a higher percentage of patients died more rapidly compared to patients with lower expression of *TMEM230* (Figures 1B,C). Our previous work demonstrated that TMEM230 regulates endothelial cell sprouting and migration associated with angiogenesis in early Zebrafish development (Carra et al., 2018) and TMEM230 protein was reported to be a component of vesicle trafficking and turnover (Kim et al., 2017; Mandemakers et al., 2017; Conedera et al., 2018; Deng et al., 2018; Wang et al., 2021). A functional enrichment analysis of the differentially expressed genes in patient derived LGG with low and high expression level TMEM230 was then performed with DAVID 6.8 and only the terms with a corrected *p*-value (Benjamini, Bonferroni or FDR) <0.05 were considered. Molecular pathways, keywords and diseases correlated with high or low levels of TMEM230 in patient gliomas derived from the functional enrichment are shown in Supplementary Tables 2-12. The analysis of genes and pathways differentially expressed support that TMEM230 has both intracellular and extracellular roles. In particular, the intracellular role is in cell membrane and extracellular matrix regulation and cell adhesion. An extracellular role was identified associated with cell cargo and exosome trafficking in all the types of gliomas analyzed from patients (Supplementary Tables 2-12, see red arrow, yellow and green highlighted terms). A representative heatmap indicates the most variable expressed genes in patient astrocytoma using the R with the “pheatmap” package (Figure 1D). To investigate the intracellular and extracellular role of TMEM230 in GBM, the U87 glioblastoma cell line that expresses TMEM230 and recapitulates the GBM invasive and proangiogenic tumor cell properties was chosen for functional analysis.

### Constitutive Inhibition of Endogenous TMEM230 Expression in U87-MG Cells

**FIGURE 2:**
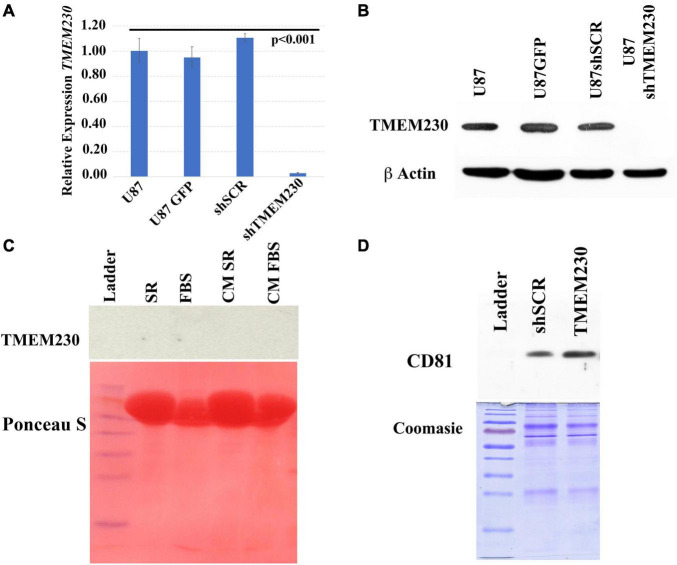
Validation of endogenous and lentivirus downregulation of *TMEM230* mRNA and protein expression in U87-MG cells. **(A)** Validation of constitutive downregulation of endogenous *TMEM230* transcript expression with lentiviral system. Endogenous control: HPRT, Error bars represent 95% confidence interval. **(B)** Validation of constitutive downregulation of endogenous TMEM230 protein expression with the lentiviral system. Endogenous control: β-actin. **(C)** Western blot analysis (top panel) showed TMEM230 protein was not detected in fetal bovine serum (FBS), serum replacement (SR) or conditioned media (CM) containing serum replacement or FBS (CM SR and CM FBS) obtained from endogenous TMEM230 expressing U87 cells. Ponceau S staining (lower panel) showed an abundance of protein was loaded. **(D)** Coomassie blue staining showed an increase of expression of extracellular vesicle membrane protein, CD81 in conditioned media of U87 cells constitutively over-expressing TMEM230 with respect to conditioned media collected from control cells (U87GFP and U87shSCR). Detection of CD81 **(D)** but lack of detection of TMEM230 protein in culture media **(C)** supports that the TMEM230 protein regulates extracellular vesicle generation or secretion but is not itself a component of extracellular vesicles.

Lentiviral constructs to constitutively upregulate and downregulate *TMEM230* mRNA and protein expression were generated. U87-MG cells were transduced with a dual promoter containing lentiviral construct expressing the copGFP reporter and *TMEM230* mRNA (for TMEM230 upregulation), with copGFP and a short hairpin sequence designed to downregulate *TMEM230* (U87shTMEM230) or with copGFP alone and copGFP with a scrambled short hairpin sequence (U87shSCR), both used as control. The effectiveness of the shTMEM230 construct in down regulating TMEM230 protein in U87 cells was verified by comparison with the U87 control cells (Figures 2A,B). Potential unwanted off-site effects of the sh-mediated downregulation of endogenous TMEM230 were previously evaluated and not observed with Tmem230 specific morpholino oligos in zebrafish (Carra et al., 2018). Fetal bovine serum (FBS) may contain bovine generated TMEM230 protein or extracellular vesicles associated with TMEM230 protein (Guida et al., 2020). Therefore, the role of the human endogenous TMEM230 protein and endogenous TMEM230 generated vesicles in U87 cells was evaluated using 2 different tissue culture conditions, in which the culture media was identical except for containing serum replacement (SR) or extracellular vesicle depleted FBS. The use of SR ensured that the assays in which TMEM230 was downregulated in U87 did not contain and was not compensated by TMEM230 protein and TMEM230 associated vesicles exogenously derived from bovine serum. Use of FBS prepared by removing extracellular vesicles also guaranteed that there were no TMEM230 associated vesicles exogenously derived from the bovine serum in the culture assays. An additional purpose of using SR containing culture conditions was to determine whether the function of TMEM230 was dependent or modified by known or unknown soluble sera components such as cytokines, growth factors and inflammation modulating factors present in FBS.

Since serum replacement, unlike FBS is composed by defined proteins artificially derived and contains no animal protein or vesicles, as expected, TMEM230 protein was not detected in SR (Figure 2C, top panel lane 2, SR). TMEM230 protein was also not detected in vesicle depleted FBS (Figure 2C top panel lane 3, FBS). While TMEM230 protein was not detected, Ponceau S staining panel (bottom) shows the considerable amount of protein loaded for each condition. While TMEM230 protein was not detected in conditioned media generated by U87 cells cultured in SR or vesicle-deplete FBS conditions (Figure 2C, top panel lanes 4 and 5), in conditioned media from cells in which TMEM230 was upregulated using a lentiviral system, increased detection of CD81 (extracellular vesicle associated marker) was observed (Figure 2D, lane 3, TMEM230), suggesting that TMEM230 is associated with vesicle generation, turnover and/or secretion (Figure 2D). Coomassie staining, bottom panel verifies equal protein loading was performed (Caby et al., 2005; Haqqani et al., 2013; Jeppesen et al., 2014).

### Constitutive Downregulation of Endogenous TMEM230 Promoted Morphological Remodeling of Cell and Cytoplasm, Cell Detachment, and Reduction in U87-MG Re-passaging Capacity

**FIGURE 3:**
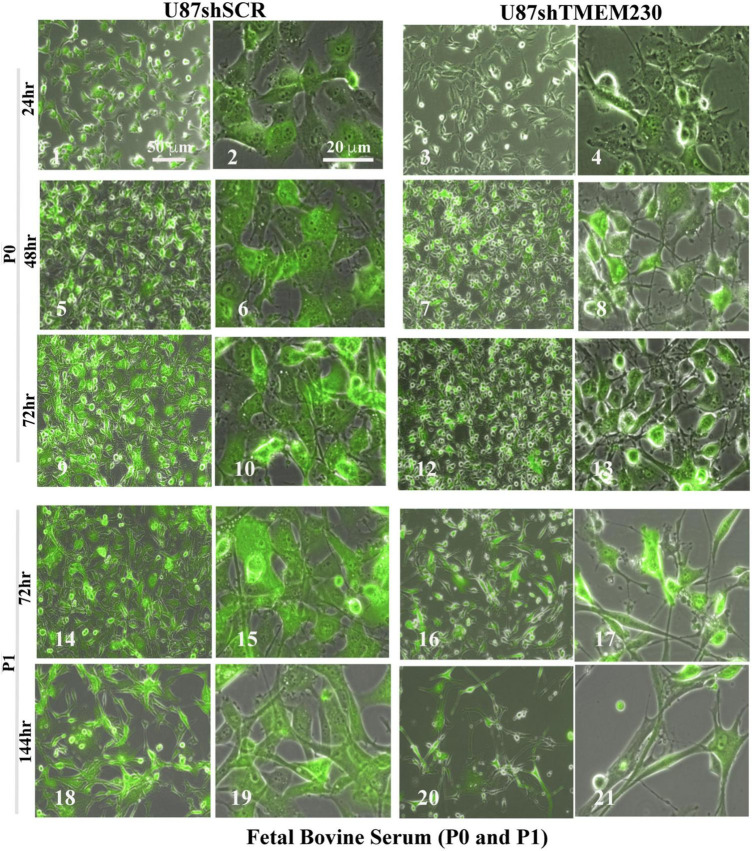
Downregulation of TMEM230 was sufficient to promote loss of U87-MG substratum adhesion capacity in fetal bovine serum containing media. Control (U87shSCR) and U87 cells in which TMEM230 was constitutively downregulated (U87shTMEM230) were cultured in extracellular vesicle depleted FBS (Fetal Bovine Serum) containing culture media. Equal number of control cells and cells in which endogenous TMEM230 was downregulated were plated (P0) in vesicle depleted FBS containing culture media and monitored over 72 h, starting from when green fluorescent protein (GFP) expression was first observed (0 h, not shown). Cells in which TMEM230 were downregulated displayed decrease in cytoplasm dimensions and disrupted cytoplasmic invadopodium like extensions. Cells were re-passaged (P1) and monitored for additional 144 h. Re-passaged cells (P1) displayed a more limited capacity for cell adhesion, supporting that TMEM230 is necessary for scaffold attachment of cells.

**FIGURE 4:**
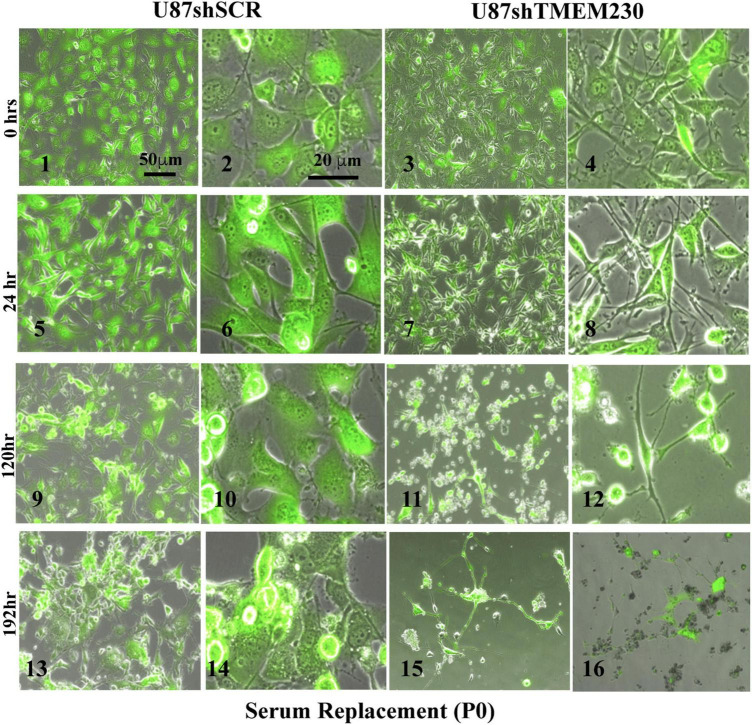
Downregulation of endogenous TMEM230 was sufficient to promote loss of U87-MG substratum adhesion capacity in serum replacement containing media. Control (U87shSCR) and U87 cells in which TMEM230 was constitutively downregulated (U87shTMEM230) were cultured in serum replacement (SR) containing media (P0). Equal number of control cells and cells in which TMEM230 was downregulated were plated in SR containing culture media and monitored over 192 h, starting from when GFP expression was first observed (0 h).

Time course experiments in which TMEM230 was constitutively down regulated revealed that U87-MG cells cultured in media containing vesicle depleted FBS (Figure 3) or SR (Figure 4) showed over time a rapid change in cell and cytoplasm morphology and a decrease in the number of cells anchored to tissue culture plates. U87shTMEM230 cell cytoplasm contracted within 24 hr in vesicle depleted FBS conditions (Figure 3, panels 3,4) or in SR conditions (Figure 4, panels 7,8) compared to control cells (Figure 3, panels 1-2, Figure 4, panels 5-6). Onset of the GFP reporter expression is at 0 hr. These results suggest loss of normal cytoskeleton structure and loss of cell membrane interaction with the extracellular scaffold with downregulation of endogenous TMEM230 protein. Change in cell morphology and fragmentation of cytoplasmic invadopodium like extensions were correlated with the loss of the anchorage ability of the cells, as observed with less cells attached to the culture plates over time see 0-72 h. See P0 for FBS (Figure 3, panels 1-13) and 0-192 h for SR (Figure 4, panels 1-16). Cells in which TMEM230 was downregulated also displayed significantly less ability to reattach with subsequent re-passaging (P1) compared to control cells expressing TMEM230 in vesicle depleted FBS. Compare 72 h in P0, Figure 3, panels 12-13, and panels 16-17. Second passage (P2) U87shTMEM230 cells in FBS displayed almost no adhesion capacity when attempts were made to generate a third passage on tissue culture plates (data not shown). In contrast, indefinite re-passaging was possible for U87shSCR control cells cultured in FBS (not shown). Green fluorescent protein control cells expressing endogenous TMEM230 cultured in SR containing media appeared more stressed (in terms of cell morphology, fragmentation of cytoplasmic extensions and substratum attachment capacity) compared to control cells cultured in FBS conditions. See SR conditions, Figure 4, panels 13, 14 at initial plating and FBS conditions, Figure 3, panels 18, 19 at P1. This was likely due to SR lacking essential factors necessary for maintaining the metabolic and growth demands of tumor cells. This suggested that TMEM230 may protect tumor cells in conditions in which essential extracellular growth factors are reduced or absent as in the case of SR media conditions. SR conditions may therefore recapitulate deficient conditions associated *in vivo* with a rapidly proliferating tumor cells in an insufficient vascularized and rapidly expanding or large tumor mass. Collectively, these analyses support that TMEM230 is necessary for U87 cells to maintain anchorage capacity to extracellular scaffolds/substratum or proper cytoskeleton function and structure. Additionally, TMEM230 may protect cells in stressful environments or conditions, such as those associated with growth factor deficiency and/or hypoxic tumor cores.

It is reported that TMEM230 is associated with vesicle generation, trafficking and turnover (Deng et al., 2016, 2018), in agreement our data suggested that increased expression of TMEM230 correlated with extracellular vesicle release (Figure 2D). We therefore evaluated both the intracellular and extracellular roles of TMEM230 in promoting 3D sprouting and migration in U87 cells, features that may recapitulate tumor cell aggressive properties associated with GBM, such as invasion and vascular mimicry. Additionally, we investigated the extracellular role of TMEM230 in promoting sprouting and migration of human umbilical vein endothelial cells (HUVECs), cultured in conditioned media generated by TMEM230 expressing U87 cells or by U87 cells in which TMEM230 was downregulated.

### TMEM230 Expressing U87-MG Cells Promote Human Umbilical Vein Endothelial Cell Sprouting, Motility and Tubule-Like Structure Formation

**FIGURE 5:**
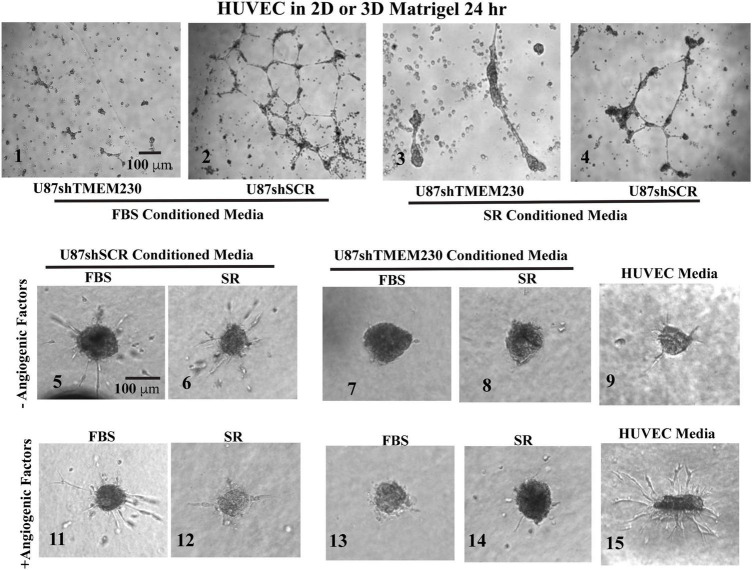
Human umbilical vein endothelial cells cultured in FBS and serum replacement containing conditioned media from U87-MG cells expressing endogenous TMEM230 promoted angiogenic behavior. **(1–4)** Representative images at 24 h of human umbilical vein endothelial cells (HUVEC) in Matrigel treated with conditioned media obtained from 3 days cultures of U87 control (U87shSCR) and U87 in which TMEM230 was down regulated (U87shTMEM230). **(5–15)** Human umbilical vein endothelial cells in 3D treated with conditioned media obtained from 3 days cultures of U87 control and U87 which TMEM230 was down regulated shown for without **(5–9)** and with angiogenic factors **(11–14)**. U87 cells were cultured in media containing fetal bovine serum (FBS) or serum replacement (SR).

To determine whether extracellular components associated with expression of TMEM230 promote blood vessel cell sprouting, HUVECs were cultured in conditioned media obtained from U87 control cells (U87shSCR) expressing endogenous TMEM230 or U87 cells in which endogenous TMEM230 expression was constitutively downregulated (U87shTMEM230). The culture media conditioned by the U87 cells was added to HUVEC plated as separated spots of confluent cells or as a 3D body formed by cell hanging drops in methylcellulose and collagen. These conditions recapitulate sprouting, migration and blood vessel formation (Nakatsu et al., 2003; Crampton et al., 2007; Nakatsu and Hughes, 2008; Heiss et al., 2015). When cultured with media conditioned by U87 cells in which TMEM230 was expressed in the absence of proangiogenic factors, HUVEC cells seeded in Matrigel formed tubule-like structures (Figure 5, panels 2,4). When cultured with media conditioned by U87 cells in which TMEM230 was downregulated, tubule structures where not observed (Figure 5 panels 1-3, compare to HUVEC cultured with conditioned media by TMEM230 U87 expressing cells, panels 2,4). Similarly, HUVEC bodies seeded in 3D (in FBS or SR media) conditioned by U87 cells in which TMEM230 was downregulated failed to display cell sprouting (Figure 5, panels 7,8) compared to HUVEC cells cultured with media conditioned by endogenous TMEM230 U87 expressing cells (Figure 5, panels 5,6).

Results support that U87 tumor cells expressing TMEM230 recapitulate blood vessel sprouting or the early steps in vessel formation without direct contact with HUVECs. Significantly less sprouting was observed in the assay in which media not conditioned by U87 was used (Figure 5, panel 9) compared to culture media conditioned by TMEM230 expressing U87 (Figure 5, panels 5,6) or when proangiogenic factors were present (Figure 5, panel 15). Surprisingly, sprouting was initiated in HUVEC cultured in conditioned media obtained from TMEM230 expressing U87 cells, regardless if proangiogenic factors were present (Figure 5, panels 11,12) or not (Figure 5, panels 5-6).

Collectively, these results support that angiogenic like behavior was initiated in HUVEC by extracellular vesicles or factors generated from U87 cells expressing endogenous TMEM230 rather than components derived from FBS or SR.

### TMEM230 Promotes U87-MG Cell Migration, Tumor and Endothelial Cell Interaction, and Displacement of Endothelial Cells

**FIGURE 6:**
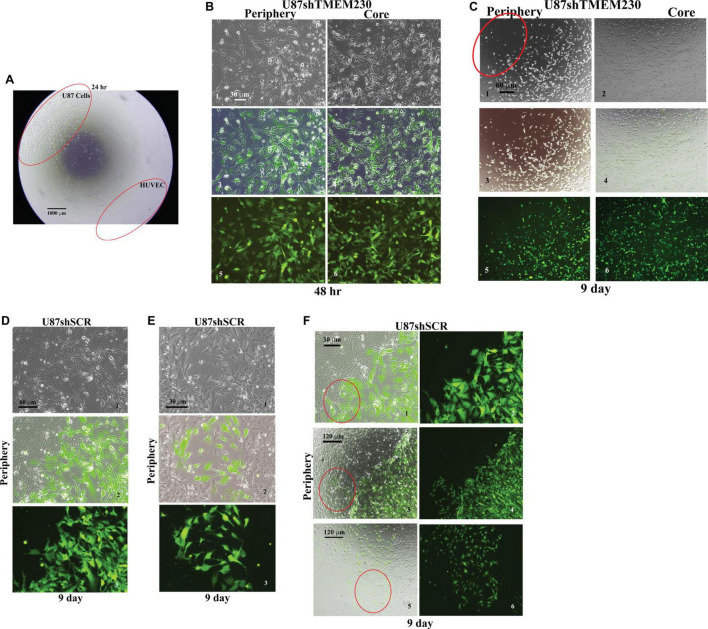
Endogenous TMEM230 promoted U87-MG cell migration, tumor-endothelial cell contact and displacement in co-culture assays. **(A)** Low magnification shows the co-culture assay set up. **(B)** Representative images showing the periphery (outgrowth) and core (initial location of cell plating) of U87shTMEM230 cells at 48 h. Downregulation of TMEM230 in U87 cells was associated with cytoplasm of reduced mass, disrupted cytoplasmic invadopodium like extensions, decreased cell anchorage and reduced contacts among initially confluent plated cells. **(C)** Representative images of U87shTMEM230 cells at periphery and core at 9 days. U87 cells with reduced TMEM230 expression displayed reduced anchorage capacity and motility (see red circle at periphery of initial site of plating of the confluent cells) compared to control cells. **(D–F)** Displacement of the confluent human umbilical vein endothelial cells by U87 control cells (U87shSCR) expressing endogenous TMEM230 through infiltration into the confluent mass of human umbilical vein endothelial cells (see red circles), a behavior that is associated with the first step of intussusceptive induced blood vessel branching.

The ability of TMEM230 to promote tumor cell migration and displacement of endothelial cell to cell contacts as an early step in blood vessel disruption was accessed by determining whether TMEM230 expressing U87 cells can invade into endothelial cells, displace endothelial cell-cell contacts and cell-ECM scaffold interactions, using co-culture assays of U87 and HUVEC cells. U87shSCR cells or U87shTMEM230 cells in parallel culture assays were plated as a confluent mass equidistant from a confluent mass of HUVECs (Figure 6A, low magnification shows assay set up). As previously observed, both peripheral and core U87shTMEM230 confluent cells showed rapid onset of aberrations in their morphologies (Figures 3, 6B). Downregulation of TMEM230 in U87 cells was associated with a cytoplasm of reduced mass, disrupted cytoplasmic invadopodium like extensions, decreased cell anchorage and reduced contacts among initially confluent plated cells (Figure 6B). Migration of U87shSCR cells or U87shTMEM230 was evaluated over time (9 days) relative to the HUVECs. U87 cells with reduced TMEM230 expression as observed in Figure 3, displayed reduced anchorage capacity and lack of motility compared to control cells (Figures 6B,C). In contrast, U87 control cells displayed extensive cell to cell contacts and motility capacity. U87 cells migrated from their initial seeding site into the core mass of non-GFP expressing HUVECs (Figure 6D-F). HUVECs, in both U87 control and U87shTMEM230 conditioned media, displayed little migration capacity for the entire time span of the assays. Contact and displacement of the confluent HUVECs were observed when U87 control cells infiltrated into the HUVEC mass, a behavior that is associated with the first step of intussusceptive induced blood vessel branching (Figures 6D-F; Djonov et al., 2003; Burri et al., 2004). The U87 assays demonstrated TMEM230 expression was necessary for tumor cell motility and infiltration-like behavior and supported that aberrant elevated levels of TMEM230 expression promote intravasation and blood vessel branching in the GBM tumor.

Collectively, contact and displacement of HUVECs observed with TMEM230 expressing U87 therefore support that TMEM230 in addition to having a role in infiltrating and remodeling tumor tissue, may also have an equally important role in tumor colonization through contact and intravasation of tumor cells into blood vessels.

### TMEM230 Dependent U87-MG Migration and Tubule Like Structure Formation Mimic Blood Vessel Formation Through Vascular Mimicry

**FIGURE 7:**
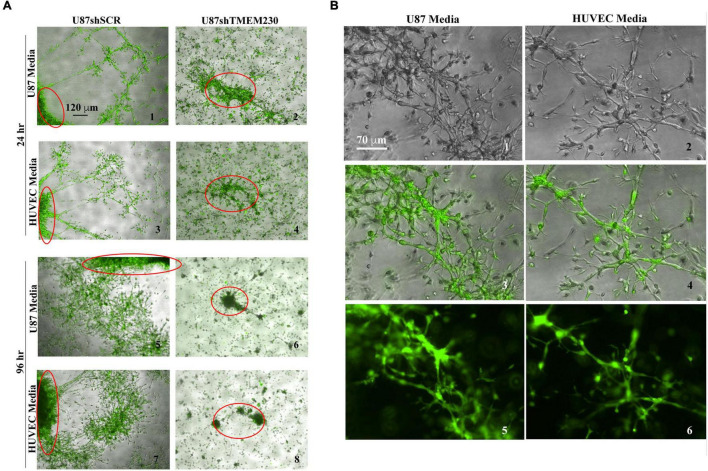
Endogenous expression of TMEM230 promoted U87-MG migration and tubule like structure formation recapitulating a vascular mimicry like behavior. **(A)** Representative 3D bodies or structures (only the borders of a larger 3D body of U87shSCR or a complete body of U87shTMEM230 cells are shown by red circles) of U87 cells expressing endogenous TMEM230 displayed vascular mimicry, cell sprouting, collective cell movement, and invasion in 3D Matrigel. U87 cells in which endogenous TMEM230 was downregulated did not generate 3D bodies of significant size in agreement that TMEM230 was required for U87 cell growth. Two different media were used for generating VM like structures from U87 cells, media used for culturing adherent U87 tumor cells or HUVEC shown in Figures 3, 5 (top panel), respectively. **(B)** Higher magnification of control cells.

Vascular Mimicry (VM) is a not completely characterized process in which tumor cells recapitulate vascularization of tumor tissue by generating microchannels or by attaching themselves to blood vessels and following their 3D structure for oxygen and nutrient effusion. VM has been identified with GBM (Arbab et al., 2015; Ahsan et al., 2017; Angara et al., 2017). Formation of blood vessel like structures by tumor cells themselves, may allow uniting tumor cell vessels with existing endothelial blood vessels to augment tissue perfusion and promote conditions suitable for metastasis formation. To evaluate whether vascular mimicry is also a property associated with TMEM230, U87 cells where plated in 3D Matrigel. In contrast to cells in which TMEM230 was downregulated, U87 cells expressing endogenous TMEM230 displayed collective movement and cell to cell contact, cell sprouting and invasion in Matrigel, generating structures reminiscent of lumen containing tubules (Figures 7A,B). These structures wholly generated and containing only tumor cells may represent the early steps of the vascular mimicry associated with GBM. Generation of 3D like vessel structures provided an additional role of TMEM230 for promoting perfusion of a tumor mass, necessary for continued aggressive tumor expansion and infiltration into tissue.

The collective results of our study support that TMEM230 promotes anchorage, motility, sprouting and branching like behavior in two diverse cell types found in GBM, tumor glial cells and resident tumor blood vessel cells, as demonstrated in U87 and HUVEC assays (Figures 3-7). Increase in expression of CD81, a marker associated with extracellular vesicles detected from conditioned media of TMEM230 expressing cells (Figure 2D), suggests that TMEM230 has both intercellular and extracellular functions and the extracellular activities are achieved through extracellular vesicles and/or secreted factors.

### RNA Sequence Analysis of Glioblastoma and Lower Grade Glial Patient Samples From the TGCA Dataset

**FIGURE 8:**
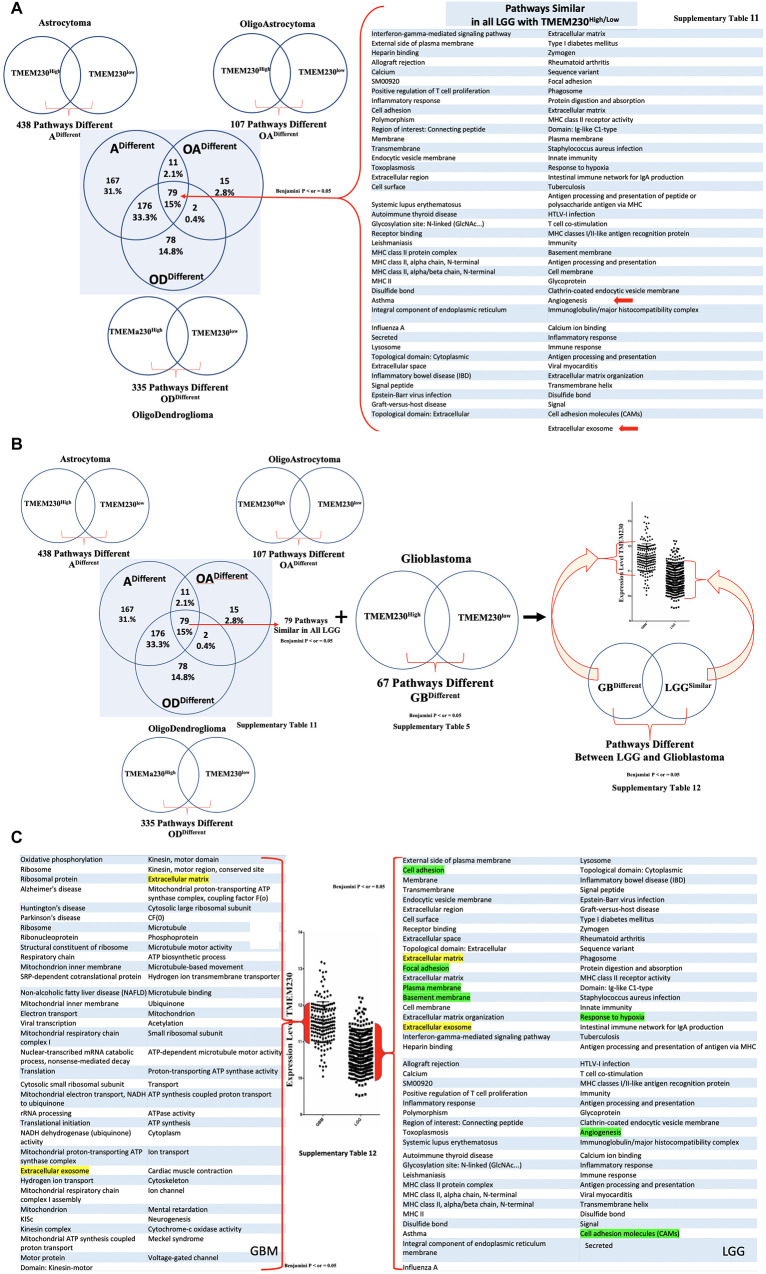
All enriched pathways identified with high or low TMEM230 expression in diverse patient glioma tumors. **(A)** Enriched pathways identified common between high or low *TMEM230* expression in all LGG of patient. **(B)** Scheme for identifying different pathways between high or low *TMEM230* expression in LGG and glioblastomas. **(C)** Enriched pathways identified different between high or low *TMEM230* expression in LGG and glioblastoma. See corresponding Supplementary Table 12.

To evaluate whether the changes in the cellular properties observed in U87 cell assays in which TMEM230 was downregulated, correlated with molecular pathways associated with patient glioma properties, differential gene expression analysis was performed from LGG subtypes and GBM tumors based on TMEM230 expression levels by comparing RNA-seq datasets. Functional enrichments with a *p*-Value (Benjamini) < 0.05 derived from the most variable expressed genes revealed 335 (oligodendrogliomas Supplementary Table 2), 107 (oligoastrocytoma Supplementary Table 3), 438 (astrocytoma Supplementary Table 4), and 67 (glioblastoma Supplementary Table 5) molecular pathways correlated with high and low levels of TMEM230 in LGG and GBM (Supplementary Figures 1-4 and Supplementary Tables 2-5). When gene expression analysis was performed comparing high-grade gliomas (GBM) with all low-grade glioma (LGG) subtypes combined and independent of the level of TMEM230 (Supplementary Figure 5 and Supplementary Table 6), pathways identified with high and low TMEM230 (Supplementary Figures 1-4) were a subset, as expected. Genes and pathways different between high grade (GBM) and low-grade gliomas (LGG) (Supplementary Figures 1-5) may provide insight into which pathways are correlated with lower overall patient survival based on tumor grading (Figure 1 and Supplementary Figures 7-9). To determine whether TMEM230 may be a master regulator in development of diverse low-grade gliomas, expression analysis was performed to identify pathways that correlated with different TMEM230 expression levels in all LGG tumors (Supplementary Figure 6 and Supplementary Table 7). Pathways were found in common in the different LGG suggesting that TMEM230 may regulate similar pathways. Candidate pathways may include signaling, extracellular matrix, cell membrane and adhesion regulation and extracellular exosome function (Supplementary Figure 6). Analysis was also performed to identify specific pathways correlated with different expression levels of *TMEM230* in low grade (G2) and high grade (G3) astrocytoma (Supplementary Figures 8, 9 and Supplementary Tables 9, 10) and high grade GBM and astrocytoma independently of the level of TMEM230 (Supplementary Figure 7 and Supplementary Table 8) and in GBM and astrocytoma, oligoastrocytoma and oligodendroglioma (Figures 8A-C and Supplementary Tables 11, 12).

Collectively, the pathways correlated with different *TMEM230* expression levels identified from LGG patient and high-grade glioma GBM support the functional cellular behavior observed in the U87 cell assays in which TMEM230 expression was modulated. In particular, downregulation of TMEM230 in LGG was associated with repression of cellular activities regulating cell membrane and extracellular matrix function and organization, cell secretion and specifically exosome activity and angiogenesis (see Figure 8A). These analyses provide strong support that TMEM230 function uncovered in U87 regulate the tumor processes of cell attachment, migration and secretion in glioma formation and progression. There was also a clear transition in changes in glial tumor cellular activity with increasing levels of TMEM230 as shown with GBM and LGG pathways being different except for exosome and extracellular matrix pathways (Figures 1A, 8B,C and Supplementary Table 12).

As the transition in gene and pathways profiles correlated with the levels of TMEM230 expression, our results support that TMEM230 is a novel clinical marker that may differentiate GBM from LGG tumor properties for application in patient diagnostics and prognosis. Surprisingly, GBM tumors with highest TMEM230 expression indicated that TMEM230 activity was associated with cellular properties correlated with the regulation of cargo transport or vesicle trafficking via ATP hydrolysis of motor proteins of the kinesin family. Collectively, expression analysis of GBM and LGG identified candidate pathways regulated by TMEM230 in glial tumor formation. These pathways include cell adhesion and migration, secretion and membrane regulation, angiogenesis, response to hypoxia, endocytic vesicle membrane, exosome vesicle regulation, lysosome and phagosome functions. Validation of candidate targets of TMEM230 activity was performed by determining whether genes identified differentially expressed in gliomas were modulated with downregulation of TMEM230 in U87 cells (Supplementary Figure 10). Representative differentially expressed genes in patient gliomas were selected for expression analysis in U87 cells (Figure 8 and Supplementary Tables 1-12). Western blot analysis indicated that SYNDECAN-1, CD44, and MOESIN proteins were downregulated with the inhibition of TMEM230 in U87 cells (U87shTMEM230) (Supplementary Figure 10A). SYNDECAN-1, CD44, and MOESIN all have roles in cytoskeleton (such as the actin family of proteins) regulation, cell adhesion and migration and tumor tissue remodeling. That TMEM230 may have a role in actin polymerization and cytoskeleton regulation was supported by immunofluorescence staining of phalloidin showing that U87 cells in which TMEM230 was downregulated displayed a decrease in long multistrand structures of actin (Supplementary Figure 10B). Immunofluorescence also showed that cell migration and scaffold regulating protein, CAVEOLIN-1 was also downregulated in U87 in which TMEM230 was downregulated (Supplementary Figure 10B). Other candidate proteins tested, such as FIBRONECTIN did not show a predicted change in expression with TMEM230 downregulation suggesting that additional genes influence glioma tumor formation and progression (Supplementary Figure 10C).

The collective analyses provide insight into how TMEM230 may regulate cellular activities in tumor tissue remodeling and aberrant vascularization in tumor development and progression. TMEM230 may be a novel candidate gene target to repress both tumor cell properties and tumor driven angiogenesis and consequently in the improvement of patient overall survival, not just for patients with high-grade glioblastoma and low-grade gliomas but also other types of abnormally vascularized tumors.

## Discussion

Tumor associated angiogenic switch is an event driven by the interactions of the tumor micro-environment and tumor cells that may lead to aggressive tumor properties and significant increase of probability tumor recurrence, subsequent to therapeutic intervention and patient mortality (Brown and Giavazzi, 1995; Bissell, 1999; Chintala et al., 1999; Bello et al., 2004; Box et al., 2010; Brooks et al., 2010; Alves et al., 2011; Belotti et al., 2011; Hielscher and Gerecht, 2012; Catalano et al., 2013; Ahir et al., 2020). While various known stimuli such as oxygen deprivation, inflammation and mechanical stress are inducers of angiogenic switch, key molecular components of the process are still not fully known. For instance, it is unclear if angiogenic switch utilizes similar genes and pathways in diverse tumors. Comprehensive characterization of tumor promoting angiogenic and extracellular matrix remodeling factors will contribute to identifying novel therapeutic targets for anticancer therapy, especially considering that existing antiangiogenic therapies based on canonical factors such as VEGF and VEGFR have proven often ineffective in certain tumors, such as GBM (Achen and Stacker, 1998; Fischer et al., 2005; Bergers and Hanahan, 2008; Argyriou et al., 2009; Crawford and Ferrara, 2009; Jo et al., 2012; Chowdhary and Chamberlain, 2013; de Groot et al., 2013; Arrillaga-Romany and Norden, 2014; Bartolotti et al., 2014; Curry et al., 2015; Ghiaseddin and Peters, 2015; Ameratunga et al., 2018; Jo and Wen, 2018; Anthony et al., 2019; Ceci et al., 2020; Funakoshi et al., 2020). Search of published and open access research on microarray, sequencing and proteomic expression analyses did not uncover any datasets to evaluate whether TMEM230 was differentially expressed specifically between non-malignant glial cells and glial cells from low- or high-grade gliomas. Surprisingly, also no patient study was available that allowed for directly comparing expression of any gene in non-malignant glial cells and glial cells from low- or high-grade gliomas. Existing gene expression studies only allowed comparing glial cells from non-malignant tissue with tumor brain tissue in toto, or specific cell types but without the non-malignant counter-part cell from single cell sequencing data (Darmanis et al., 2015, 2017; Batiuk et al., 2017, 2020; Bayraktar et al., 2020). While studies do not allow a direct comparison of TMEM230 between non-malignant and malignant specific cell types of the brain, they support that TMEM230 is ubiquitously expressed in most cell types from the human non-malignant or malignant brain of patients, including glial cell lineages (see Supplementary Figure 11).

Our study here supported that TMEM230 promoted angiogenesis by inducing sprouting and tubule-like structures in HUVECs and vessel like structures by tumor cells themselves through a process described as vascular mimicry. Additionally, when TMEM230 was down regulated in tumor cells, the tumor cells lost the ability for substratum adhesion and consequently, substrate dependent motility. Control tumor cells expressing endogenous TMEM230 displayed significant migration capacity and when confronted with endothelial cells in their path, infiltrated, enveloped or displaced confluent colonies of endothelial cells, suggestive of the intussusceptive structural remodeling of blood vessels, leading to new vessel branching. TMEM230 appears to have the capacity to augment tissue vascularization by 3 known mechanisms that promote oxygen and nutrient diffusion of tissue. One is migration and homing like behavior of tumor cells to existing blood vessels. These properties allow tumor cells to home to, infiltrate and displace endothelial cells resulting in the generation of new branching structures by intussusceptive structural remodeling of existing blood vessels (Figure 6). Another is tumor cells expressing proangiogenic paracrine factors or secreting vesicles that co-opt epigenomic mechanisms of endothelial cells. The secreted factors induce new sprouting and vessel like structure formation of endothelial cells (Figure 5). The last mechanism is tumor cells remodeling the microenvironment to generate microchannels or vessel like structures that recapitulate lumen formation, a process described as VM (Figure 7). The 3 different models of tissue vascularization are well characterized in glioblastoma tumors. These models are described in diverse vascularized tumors (Burri et al., 2004; Djonov and Makanya, 2005; Dome et al., 2007; Hillen and Griffioen, 2007; Makanya et al., 2009; Sacewicz et al., 2009; Hlushchuk et al., 2011; Axnick and Lammert, 2012; De Spiegelaere et al., 2012; Ribatti and Crivellato, 2012; Ribatti and Djonov, 2012; Mentzer and Konerding, 2014; Bugyik et al., 2016; Krishna Priya et al., 2016; Nowak-Sliwinska et al., 2018; Diaz-Flores et al., 2020; Saravanan et al., 2020; Ribatti and Pezzella, 2021). Tumor cells generating microchannels through degradation and remodeling of the tumor extracellular matrix recapitulates lumen formation associated with neovascularization. Microchannels allow tumor cells to directly interact with existing and distant blood vessels. Consequently, the microchannels allow for increased passive oxygen and nutrient diffusion. Tumor cells may also follow along the blood vessels, whereby the blood vessel structures act as guides, allowing tumor cells to spread both internally into and externally around existing blood vessels (Ge and Luo, 2018; Zavyalova et al., 2019; Fathi Maroufi et al., 2020; Mei et al., 2020; Wechman et al., 2020; Zhang et al., 2020; Majidpoor and Mortezaee, 2021; Ribatti and Pezzella, 2021; Treps et al., 2021; Wei et al., 2021).

Insight into the molecular components of these 3 mechanisms is provided by gene expression analysis obtained from patients with glial tumors (Supplementary Figures and Tables) and the observation that expression of TMEM230 in GBM is positively correlated with increased expression of genes and pathways associated with extracellular vesicles, angiogenesis, cell adhesion and motility. While the TMEM230 expression profile of patient GBM was due to the contribution of diverse cell types comprising the tumor, we demonstrated that TMEM230 promoted anchorage, motility, sprouting and branching like behavior in two diverse cell types found in GBM, the tumor glial cells and resident tumor blood vessel cells, as demonstrated in U87 and HUVEC assays. Therefore, the pathways enriched in GBM in which TMEM230 expression was elevated indicated that angiogenic switch associated with GBM was a process driven by the physical interactions of different cell types and scaffold or soluble factors present in the tumor environment. This is in agreement with the identification that TMEM230 has both intracellular and extracellular activity in tumor development and tumor driven angiogenesis. As our previous results showed that TMEM230 was expressed in diverse human tumor cell lines and patient tumor cells, this was suggestive that different tumors may utilize similar TMEM230 modulated genes and pathways for promoting tumor associated angiogenic switch. As extracellular vesicles such as exosomes are known to induce angiogenesis and modulate remodeling of the tumor microenvironment, future study will need to be performed to validate the role of TMEM230 in exosome activity in angiogenesis and determine whether exosomes are from diverse TMEM230 expressing cells in GBM.

Cell adhesion molecules such as integrins and other membrane proteins, are essential for cell attachment to basement membrane and extracellular matrix components to allow migration and remodeling of the microenvironment of both tumor cells and endothelial cells (Balkwill, 2003; Levin, 2005; Mackay, 2008; Alves et al., 2011; Lechertier and Hodivala-Dilke, 2012; Armento et al., 2017; Angelopoulou and Piperi, 2018; Lefranc et al., 2018). In this study, we demonstrated that downregulation of TMEM230 inhibited the adherence of U87 tumor cells both to the basement membrane like scaffold (Matrigel, cellulose or collagen) and to polystyrene surface of tissue culture plates. Cellular migration is dependent on the transfer of force from the cytoskeleton scaffold to the ECM. Loss of adherence is correlated with inability of the tumor cells to migrate and interact with HUVECs, conditions necessary for intussusceptive structural remodeling of blood vessels and for vascular mimicry. Gene expression analysis of patients supported that expression of genes associated with integrin mediated signaling and binding, focal adhesion complex formation, extracellular and transmembrane protein turnover are well represented and correlated with increased expression of TMEM230. Gene expression analysis of patients further suggested that TMEM230 played a role maintaining tumor cell adherence to the extracellular scaffold, necessary for tumor cell motility and invasion. In agreement, endogenous inhibitors of angiogenesis are often associated with extracellular matrix or basement membrane proteins which function to interfere with endothelial cell sprouting, migration and tube morphogenesis and down regulate genes expressed in endothelial cells (Pozzi and Zent, 2009; Box et al., 2010; Simon-Assmann et al., 2011).

Gene expression analysis uncovered specific pathways associated with the increase of TMEM230 expression in LGG and glioblastoma from patients (Figure 8C and Supplementary Table 12). These pathways involved: cell adhesion, secretion and membrane regulation, angiogenesis, response to hypoxia, endocytic vesicle membrane and exosome vesicle regulation and lysosome and phagosome activities. Highest levels of TMEM230 were correlated in glioblastoma with ribosome generation, mitochondria ATP synthesis, kinesin motor proteins, ATP synthesis and ATP dependent microtubule motor activity. Kinesins are motor proteins that move large proteins, vesicles, structures and organelles such as mitochondria with ATP dependent hydrolysis along microtubules (Ali and Yang, 2020; Furnish and Caino, 2020; Konjikusic et al., 2021). Most kinesins regulate transport from intracellular locations toward the cell periphery such as membrane components for cell membrane homeostasis, turnover and recycling. These intracellular cargos in turn can be derived from phagosomes and destined for lysosome activity. Formation of large multicomponent structures such as ribosomes require motor proteins to be transported to sites of assembly. Extracellular secretion of signaling products and extracellular matrix remodeling factors such as metalloproteinases are also regulated by kinesins in normal and disease development. This activity is also coordinated with actin for cell migration and 3D structure sprouting via membrane component turnover and regeneration (Feiguin et al., 1994; Langford, 1995; Oosawa, 1995; Hehnly and Stamnes, 2007). Therefore, the results of our analysis suggest that further investigations of kinesins and additional ATP-dependent cytoskeletal regulators may be worthwhile in the context of TMEM230 function.

In conclusion, the tumor properties associated with glioma patients are supportive of the functional tumor role of TMEM230 demonstrated in the U87 and HUVEC assays performed in this study. Higher levels of TMEM230 promoted aggressive tumor behavior, remodeling and increased endothelial and tumor cell (vascular mimicry) based vascularization of 3D scaffolds through intracellular and extracellular activities of TMEM230. All evidence supports that TMEM230 may be a novel target gene for both anticancer and anti-angiogenesis for certain highly vascularized tumors that are currently intransigent to therapeutic interventions. Moreover, molecular tumor and angiogenic pathways identified with TMEM230 may help develop novel therapeutic strategies for inhibiting migration, abnormal tumor microenvironment and blood vessel remodeling of tumor glial cells, and tumor driven angiogenesis of glioblastoma cells. Moreover, this study combined with our previous research supports that precision regulation of TMEM230 epxression levels in patients may likely promote normalization of tumor formed abnormal blood vessels to allow for better delivery of antitumor therapeutic drugs.

Inhibition of endogenous angiogenic promoting factors, such as TMEM230, are attractive targets for cancer therapy and tumor associated angiogenesis, as they may be less toxic and less likely to lead to drug resistance than exogenous inhibitors. Since we have previously demonstrated that TMEM230 appears to be a master regulator of angiogenesis, independent and in parral to the VEGF and NOTCH signaling pathways, our study presents a novel strategy and alternative target for inhibiting the VEGF dependent angiogenic pathway. This is especially relevant in clinical cases where VEGF specific targeted or antibodies therapies do not function. Modulation of TMEM230 may have applications in addition to cancer treatment, for instance disorders in which unregulated angiogenesis results in unwanted new blood vessel formation such as in macular degeneration.

## Data Availability Statement

The raw data supporting the conclusions of this article will be made available by the authors, without undue reservation.

## Author Contributions

CC performed all the U87 and HUVEC experiments, acquired the microscope images, and analyzed all the data. VMg conceived and designed experiments, provided guidance, and analyzed all the data. EA performed all the bioinformatics analysis of the TCAC and TACG data for glioblastomas and lower grade glial tumors. PP, VMr, and LV generated lentiviral construct for TMEM230 modulation. PP performed RNA extraction and expression analysis from U87. EP, RG, DMz, and MP contributed ideas. AC, GG, EM, and SL contributed to image analysis. CD, GL, and GD contributed with different tools. SM, GN, JK, and BG contributed ideas and tools. AN, DMr, and FB provided valuable guidance. GB and ID’A made critical observations. MG was a major contributor in revision of the manuscript. IZ and RR conceived, designed, optimized, jointly supervised all of the biological experiments of the project and analyzed all the data, and wrote and revised the manuscript. All authors contributed to the article and approved the submitted version.

## Conflict of Interest

IZ and RR have a patent accepted concerning the use of Agents that modulate TMEM230 in tumor associated angiogenesis. Patent Application International Publication number: 20200247882. Agents that modulate TMEM230 as angiogenesis regulators and that detect TMEM230 AS markers of metastasis. The remaining authors declare that the research was conducted in the absence of any commercial or financial relationships that could be construed as a potential conflict of interest.

## Publisher’s Note

All claims expressed in this article are solely those of the authors and do not necessarily represent those of their affiliated organizations, or those of the publisher, the editors and the reviewers. Any product that may be evaluated in this article, or claim that may be made by its manufacturer, is not guaranteed or endorsed by the publisher.

## Footnotes

1
https://bioconductor.org/packages/release/bioc/html/DESeq2.html

2
http://www.cancergenome.nih.gov/

## Supplementary Material

Supplementary Figure 1 Representative enriched pathways identified from differentially expressed Genes in patient derived oligoastrocytoma with high and low *TMEM230* expression.

Supplementary Figure 2 Representative enriched pathways identified different between high or low *TMEM230* expression from differentially expressed genes (DEG) in patient samples of oligoastrocytoma.

Supplementary Figure 3 Representative enriched pathways identified different between high or low *TMEM230* expression from differentially expressed genes (DEG) in patient samples of astrocytoma.

Supplementary Figure 4 Representative enriched pathways identified different between high or low *TMEM230* expression from differentially expressed genes (DEG) in patient samples of glioblastoma.

Supplementary Figure 5 Representative enriched pathways identified different between glioblastoma and LGG from DEG.

Supplementary Figure 6 Representative enriched pathways identified different between high or low *TMEM230* expression from DEG in LGG.

Supplementary Figure 7 Representative enriched pathways identified different between high or low *TMEM230* expression from DEG in GBM and astrocytoma.

Supplementary Figure 8 Representative enriched pathways identified different between high or low *TMEM230* expression from DEG in low grade (G2) astrocytoma.

Supplementary Figure 9 Representative enriched pathways identified different between high or low *TMEM230* expression from DEG in high grade (G3) astrocytoma.

Supplementary Figure 10 Protein validation of TMEM230 candidate responding genes in U87-MG. **(A)** Western blot analysis for SYNDECAN-1, CD44, and MOESIN proteins in U87 control cells (U87shSCR) and in U87 cells in which TMEM230 was down regulated (U87shTMEM230). Endogenous control: β-ACTIN. **(B)** Immunofluorescence analysis for CAVEOLIN-1 and ACTIN through phalloidin interaction in U87 control and shTMEM230 transduced cells (GFP staining). Nuclei are visualized with DAPI. That TMEM230 may have a role in actin polymerization and cytoskeleton regulation is supported by immunofluorescence staining of PHALLOIDIN showing that U87 cells in which TMEM230 was downregulated was associated with decrease in the number and quality in the long multistrand structures of actin. **(C)** Immunofluorescence analysis for FIBRONECTIN in U87 control and shTMEM230 transduced cells (GFP staining). Nuclei are visualized with DAPI. Expression analysis of patient samples suggests that *Fibronectin* is differentially expressed between gliomas and non-malignant glial cells. Immunofluorescence analysis supports that FIBRONECTIN is not differentially expressed in U87 control and shTMEM230 transduced cells, and therefore TMEM230 does not regulate FIBRONECTIN expression.

Supplementary Figure 11 TMEM230 expression in diverse cell types from non-malignant and malignant human brain. **(A)** Distribution of TMEM230 transcripts in different non-malignant cell populations from human brain according to the study of Darmanis (2015) (Accession: GSE67835, survey of human brain transcriptome diversity at the single cell level). Candidate cell populations were identified representative of the major neuronal, glial and vascular cell types according to published candidate gene markers. The distribution is represented by a boxplot with a minimum, the first quartile, the sample median, the third quartile and the maximum of the expression for each cell population, from the bottom to the top. The distribution profiles support that TMEM230 is expressed in most non-malignant cells at relatively equal but low levels. **(B–D)** Expression analysis of Tmem230 from Single-Cell Rna-Seq Analysis of Infiltrating Neoplastic Cells at the Migrating Front of Human Glioblastoma. **(B)** 2D-tSne representation of all single cells included from the study of Darmanis (2017) with a sample size of n = 3,589. Cell clusters are differentially colored and identified as distinct cell classes. **(C)** Expression of characteristic cell-type-specific genes overlaid on the 2D-tSne space. **(D)** Quantification of Tmem230-positive cells of distinct cell classes.

Supplementary Table1 Patient clinical features include gender, age, tumor size in cm^3^ and *TMEM230* expression level.

Supplementary Table 2 All enriched pathways identified different between high or low TMEM230 from differentially expressed genes (DEG) in patient samples of oligodendrogliomas A).

Supplementary Table 3 All enriched pathways identified different between high or low TMEM230 expression in oligoastrocytoma of patients from DEG.

Supplementary Table 4 All enriched pathways identified different between high or low TMEM230 expression in astrocytoma of patients from DEG.

Supplementary Table 5 All enriched pathways identified different between high or low TMEM230 expression in glioblastoma of patients from DEG.

Supplementary Table 6 All enriched pathways identified different between glioblastoma and LGG from DEG.

Supplementary Table 7 All enriched pathways identified different between high or low TMEM230 expression in LGG from DEG.

Supplementary Table 8 All enriched pathways identified different between high or low TMEM230 expression in GBM and astrocytes from DEG.

Supplementary Table 9 All enriched pathways identified different between high or low TMEM230 expression in low grade (G2) astrocytoma of patients from DEG.

Supplementary Table 10 All enriched pathways identified different between high or low TMEM230 expression in high grade (G3) astrocytoma of patients from DEG.

Supplementary Table 11 All pathways identified common between high or low TMEM230 expression in all LGG of patients from DEG.

Supplementary Table 12 All pathways identified different between high or low TMEM230 expression in LGG and glioblastomas from DEG.
