# Supplementary figures and images for: Transmembrane Protein TMEM230, a Target of Glioblastoma Therapy
Source: Front Cell Neurosci. 2021 Nov 17;15:703431. doi: 10.3389/fncel.2021.703431 (PMC8636015; doi:10.3389/fncel.2021.703431)

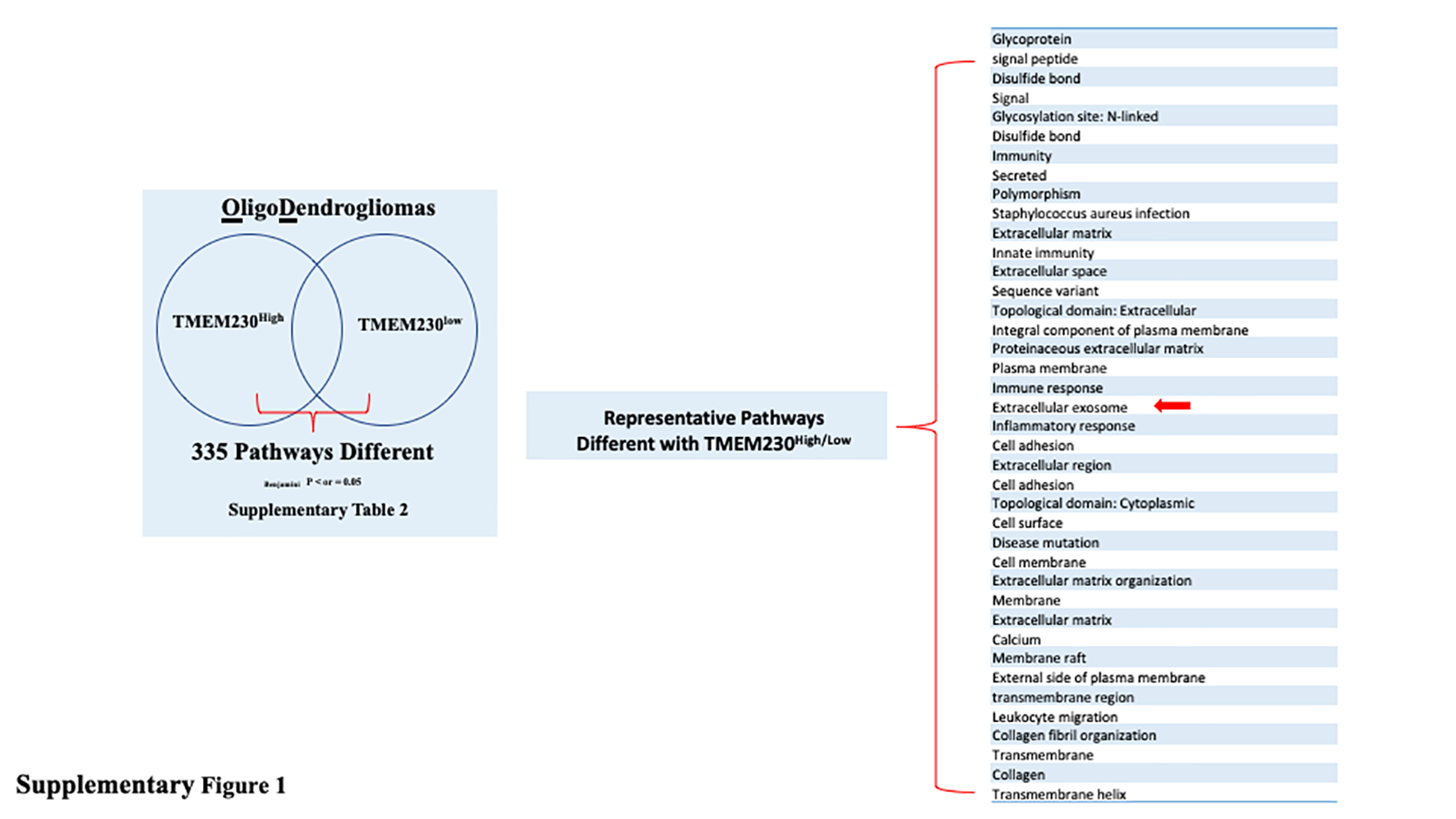

Supplement: Supplementary Figure 1 — Representative enriched pathways identified from differentially expressed Genes in patient derived oligoastrocytoma with high and low TMEM230 expression. [file Image_1.TIFF]

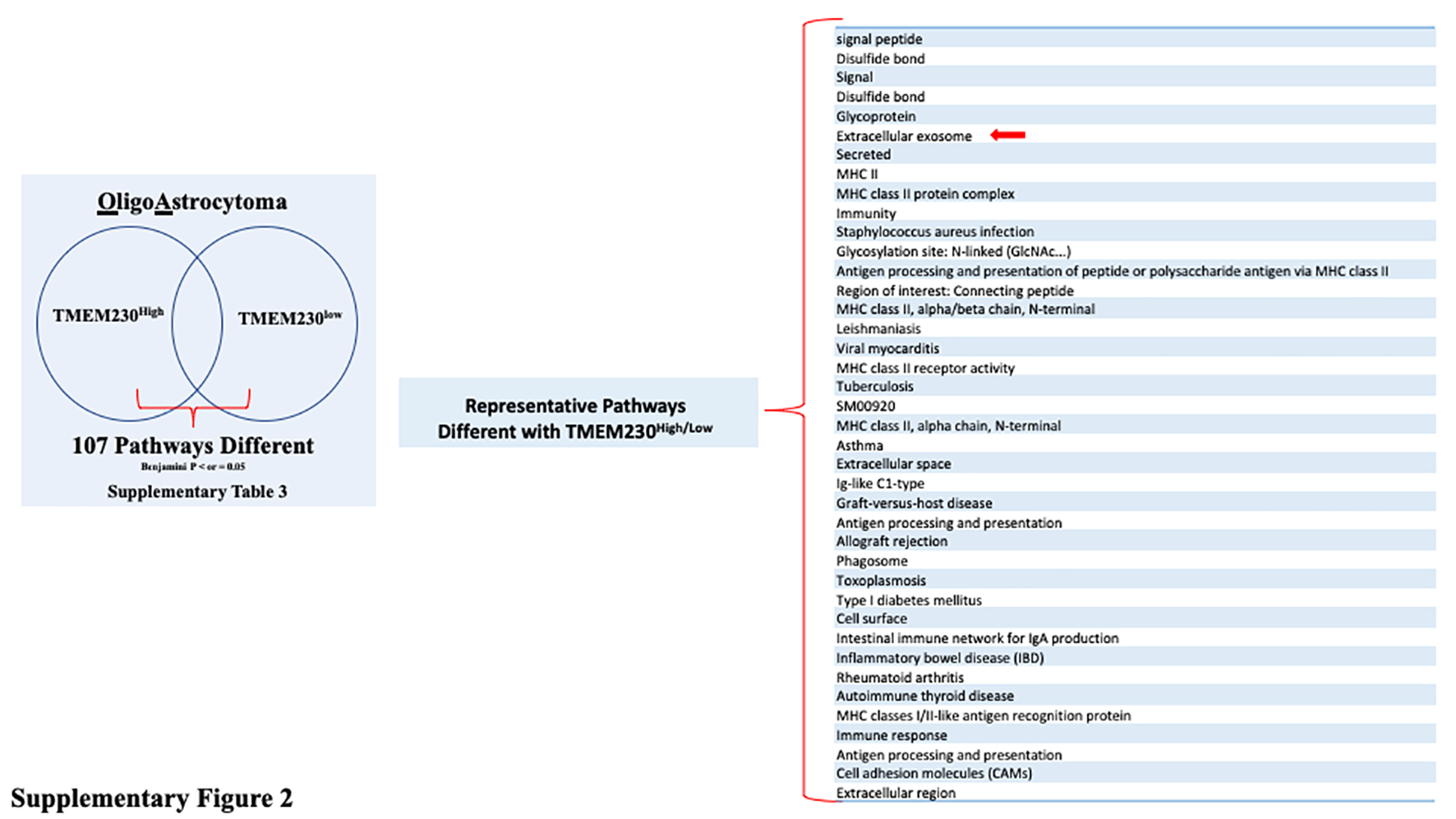

Supplement: Supplementary Figure 2 — Representative enriched pathways identified different between high or low TMEM230 expression from differentially expressed genes (DEG) in patient samples of oligoastrocytoma. [file Image_2.TIFF]

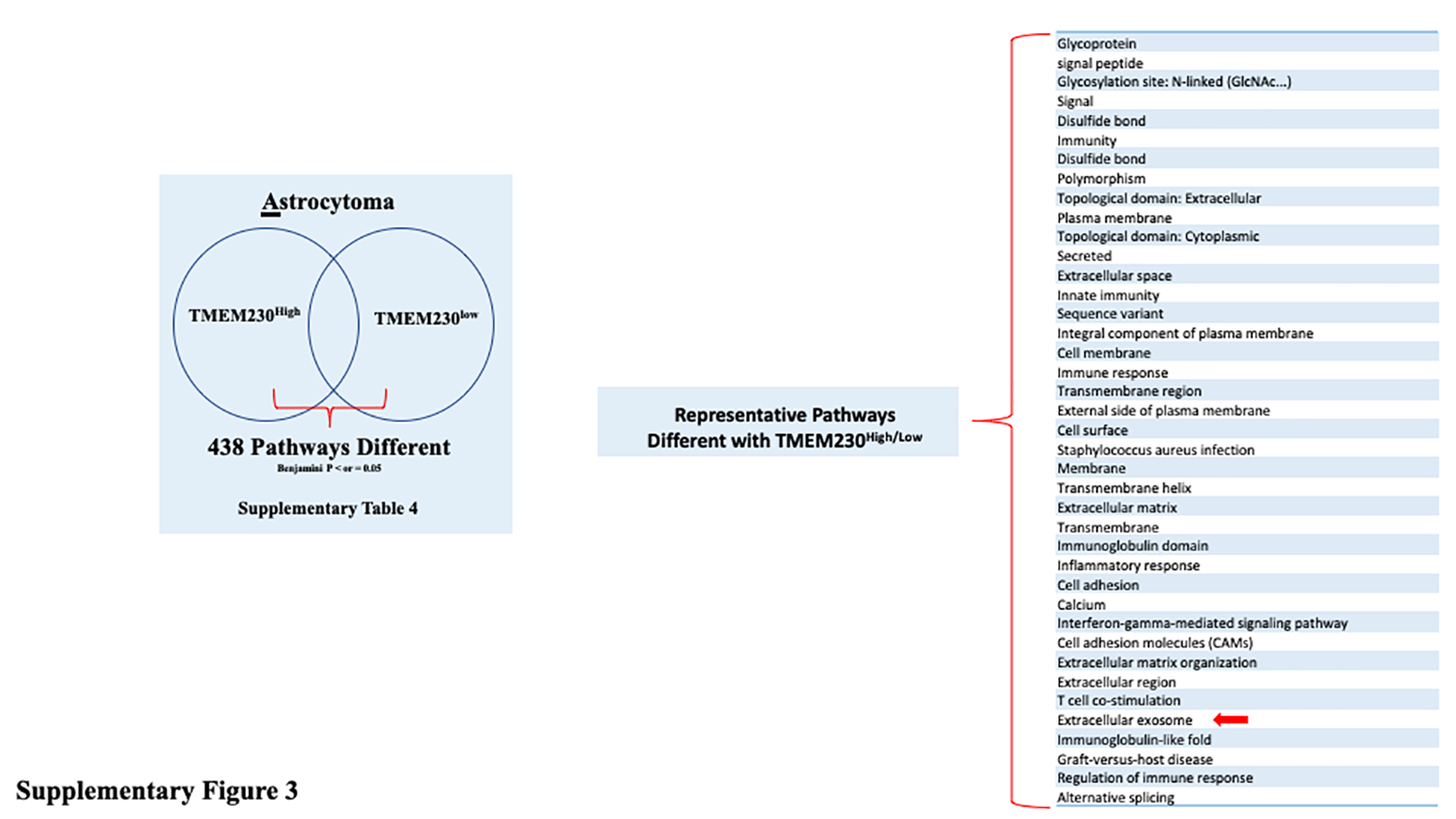

Supplement: Supplementary Figure 3 — Representative enriched pathways identified different between high or low TMEM230 expression from differentially expressed genes (DEG) in patient samples of astrocytoma. [file Image_3.TIFF]

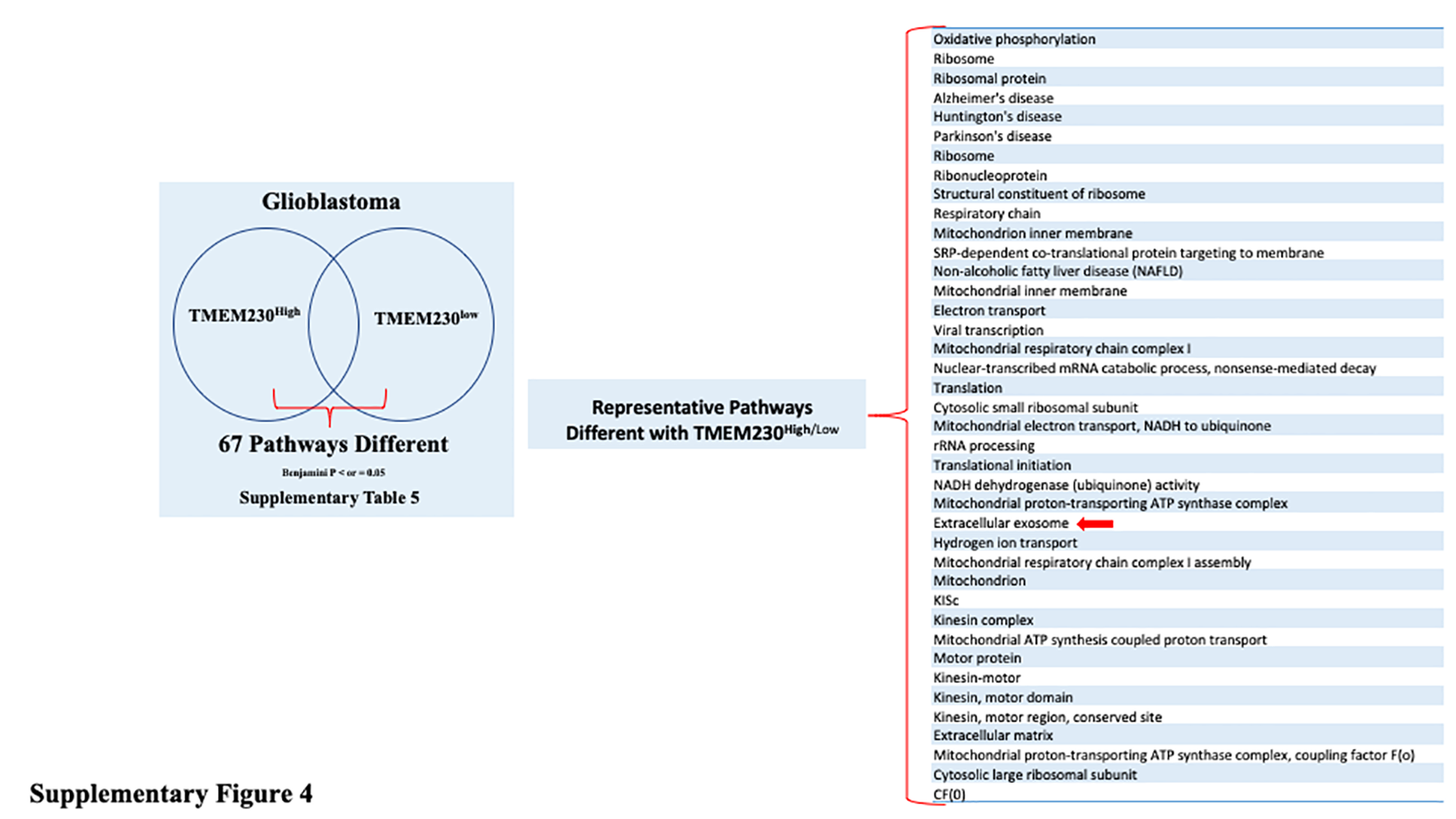

Supplement: Supplementary Figure 4 — Representative enriched pathways identified different between high or low TMEM230 expression from differentially expressed genes (DEG) in patient samples of glioblastoma. [file Image_4.TIFF]

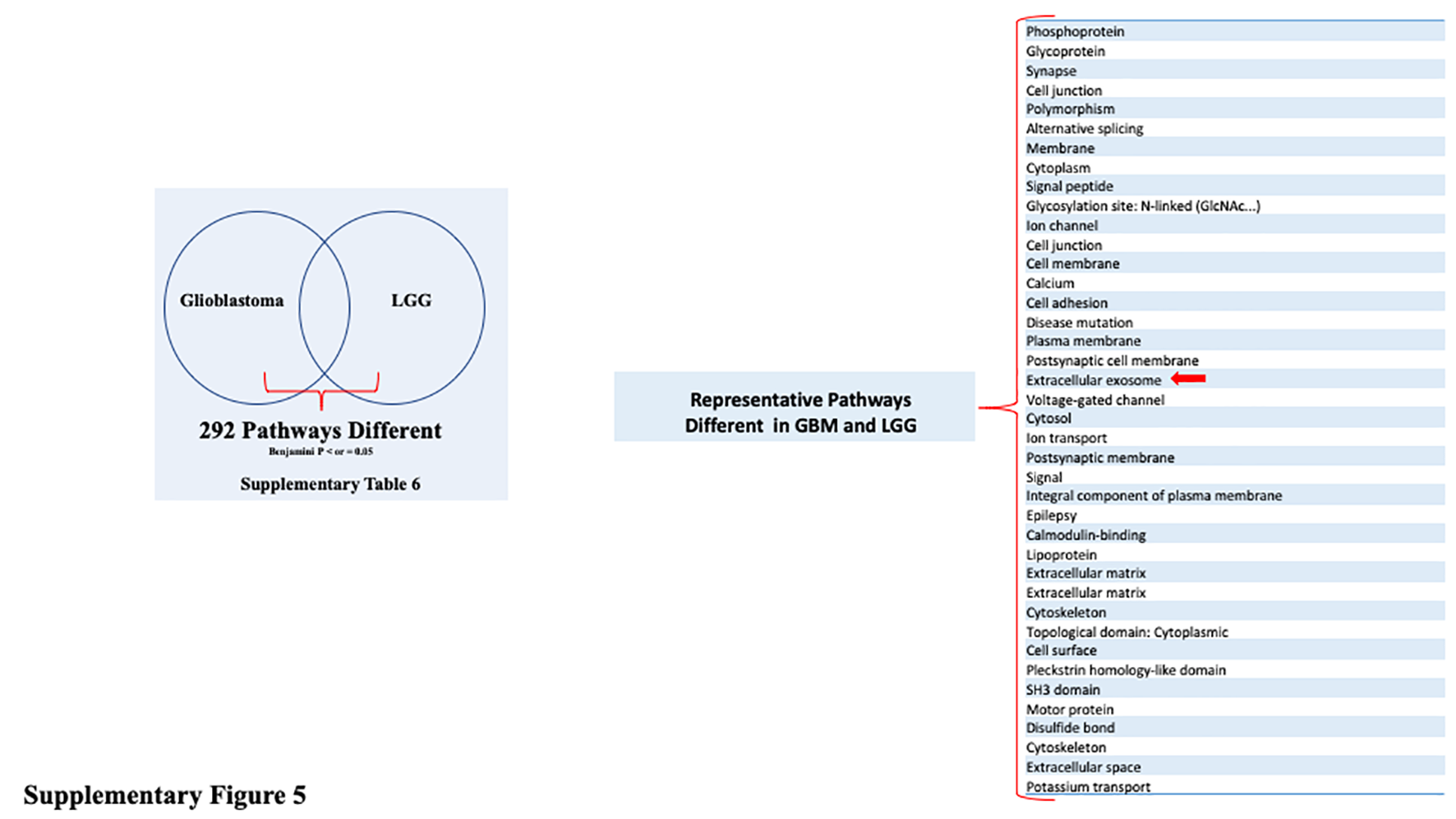

Supplement: Supplementary Figure 5 — Representative enriched pathways identified different between glioblastoma and LGG from DEG. [file Image_5.TIFF]

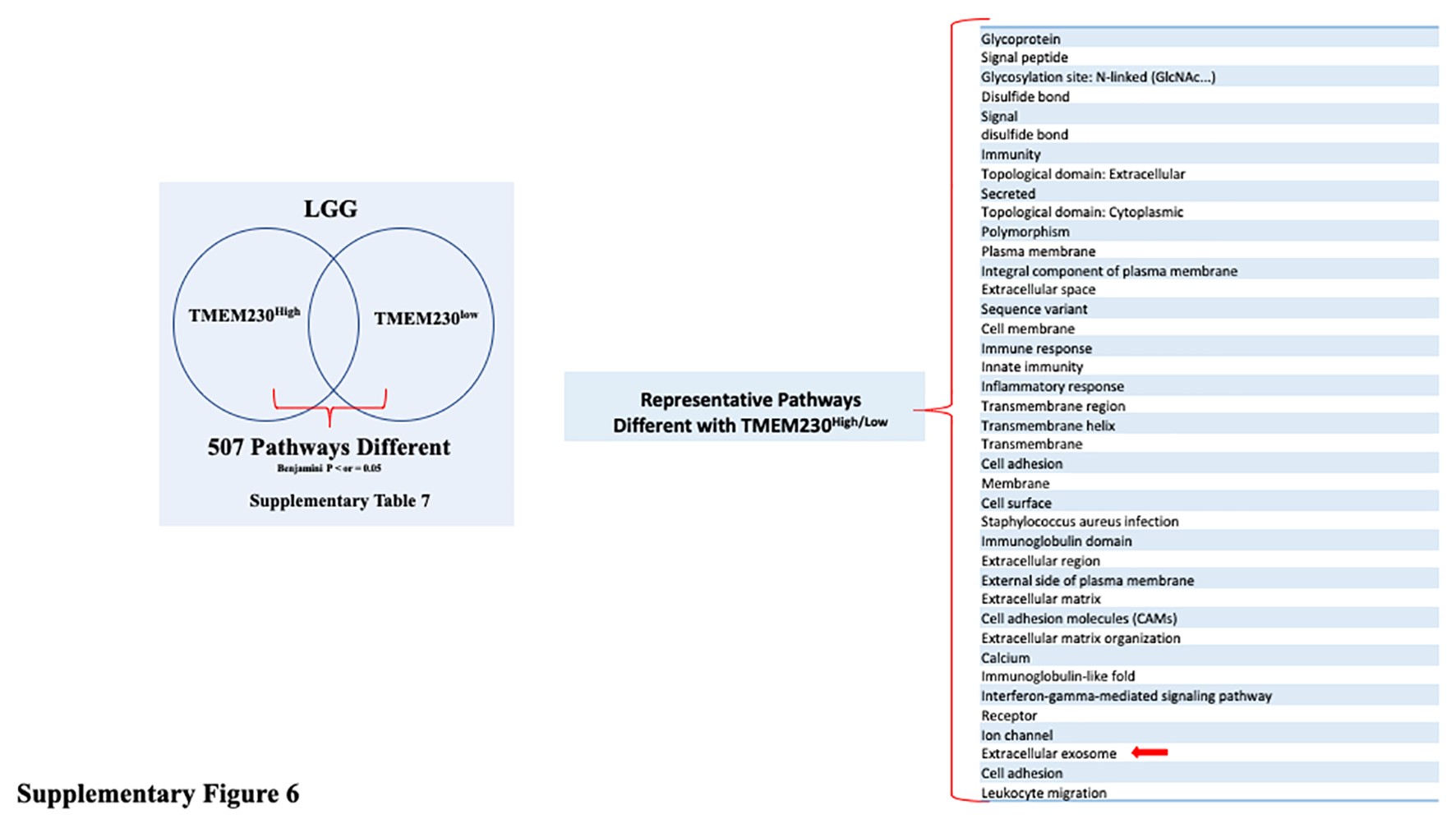

Supplement: Supplementary Figure 6 — Representative enriched pathways identified different between high or low TMEM230 expression from DEG in LGG. [file Image_6.TIFF]

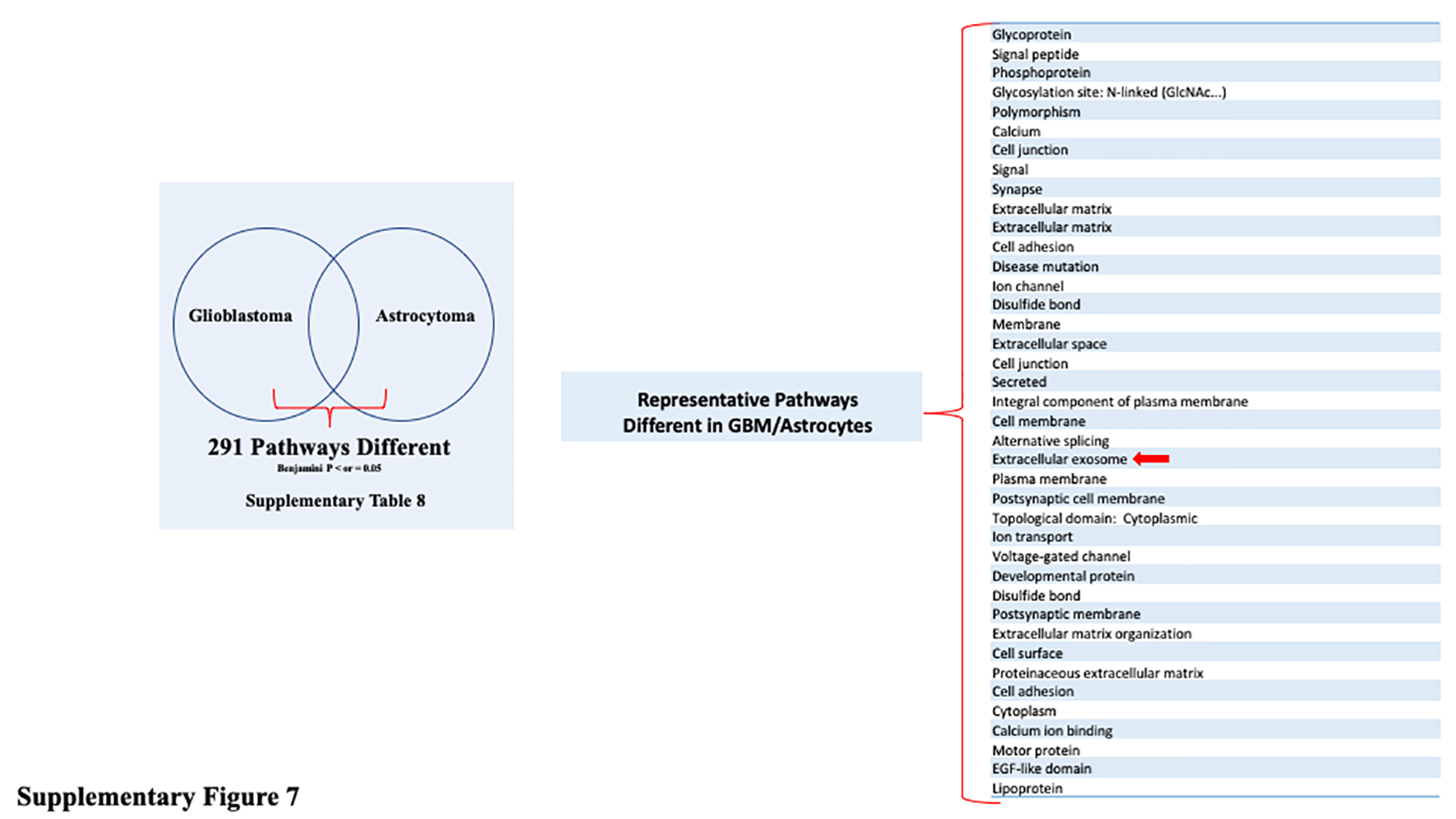

Supplement: Supplementary Figure 7 — Representative enriched pathways identified different between high or low TMEM230 expression from DEG in GBM and astrocytoma. [file Image_7.TIFF]

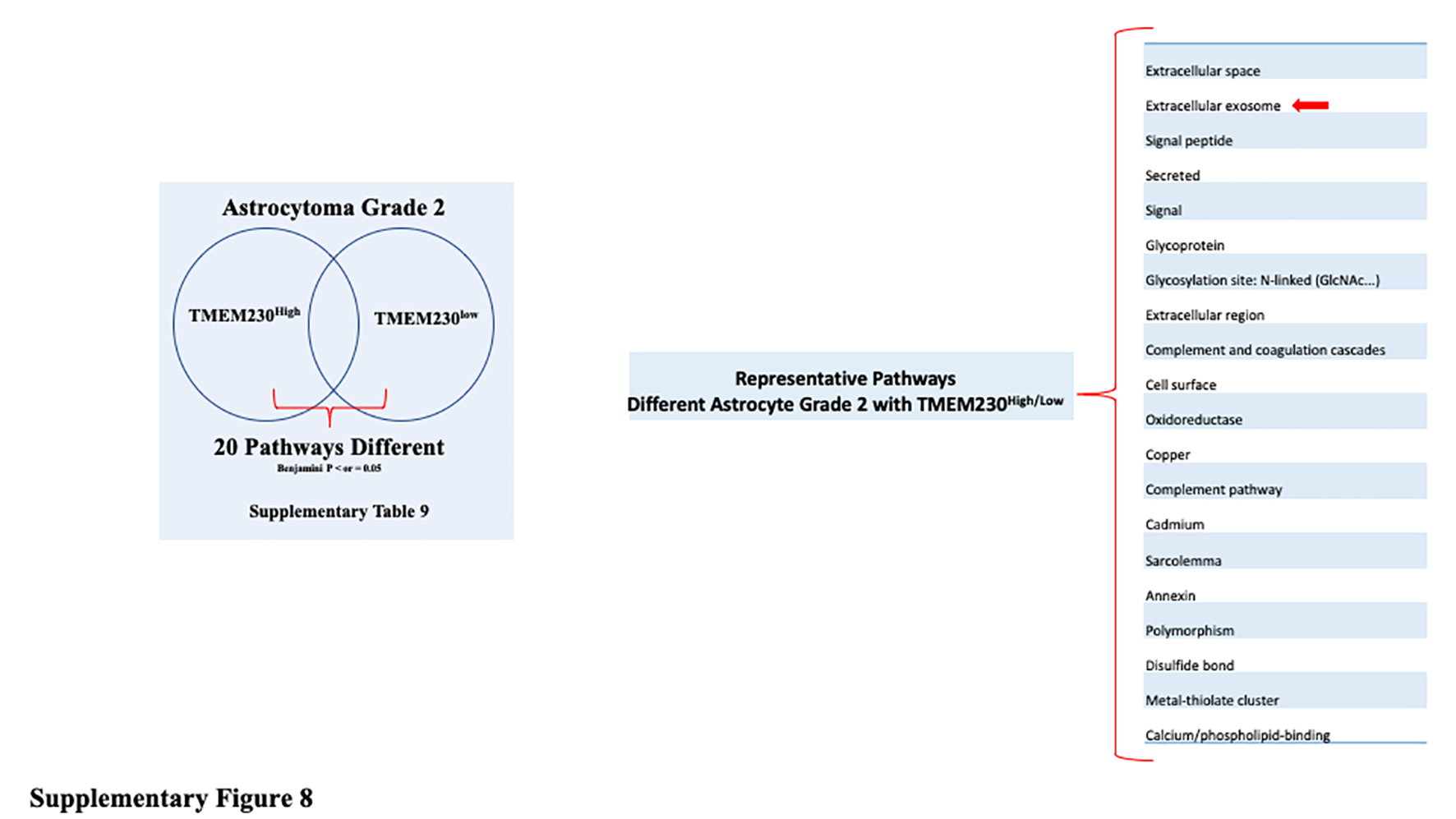

Supplement: Supplementary Figure 8 — Representative enriched pathways identified different between high or low TMEM230 expression from DEG in low grade (G2) astrocytoma. [file Image_8.TIFF]

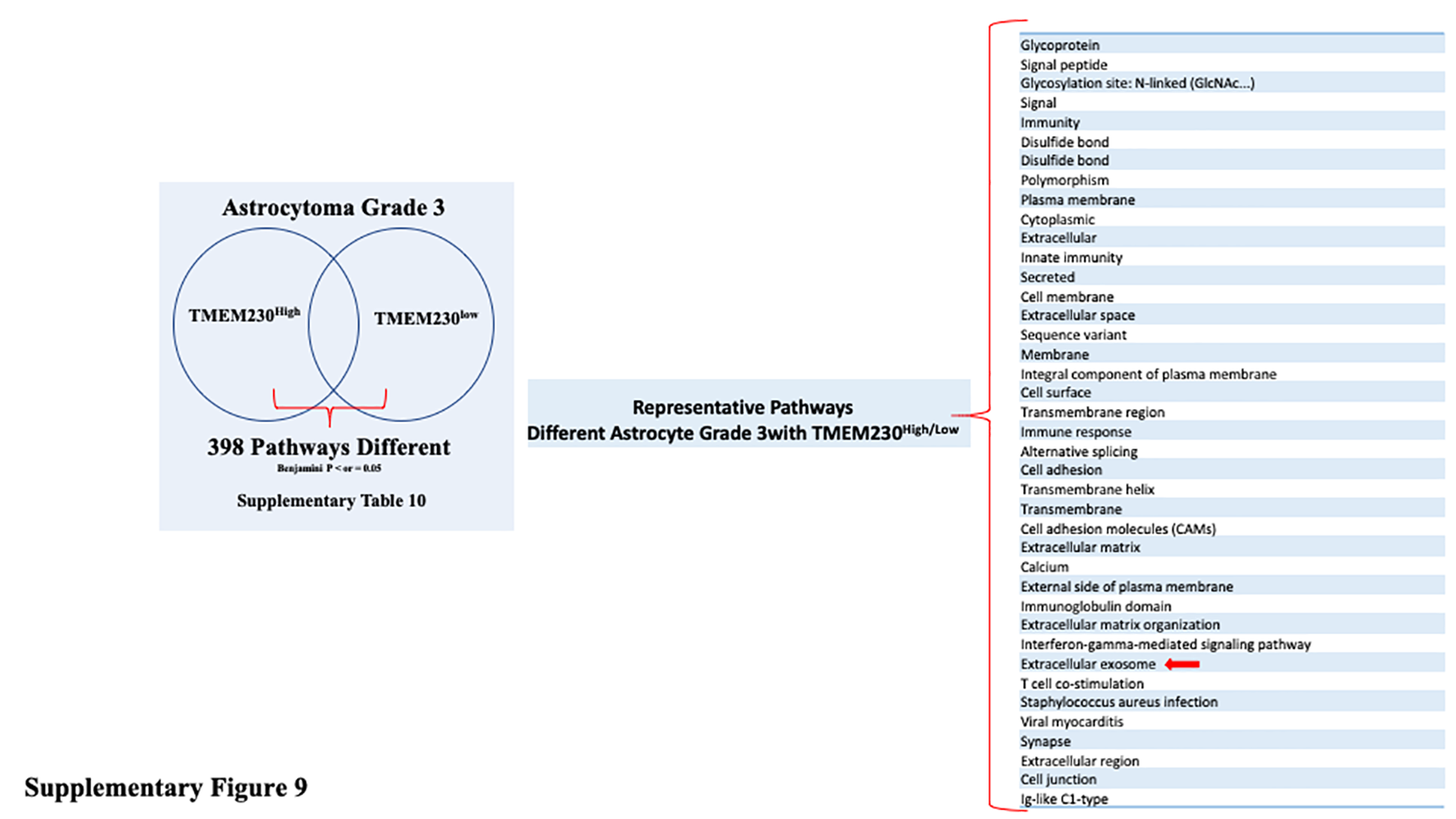

Supplement: Supplementary Figure 9 — Representative enriched pathways identified different between high or low TMEM230 expression from DEG in high grade (G3) astrocytoma. [file Image_9.TIFF]

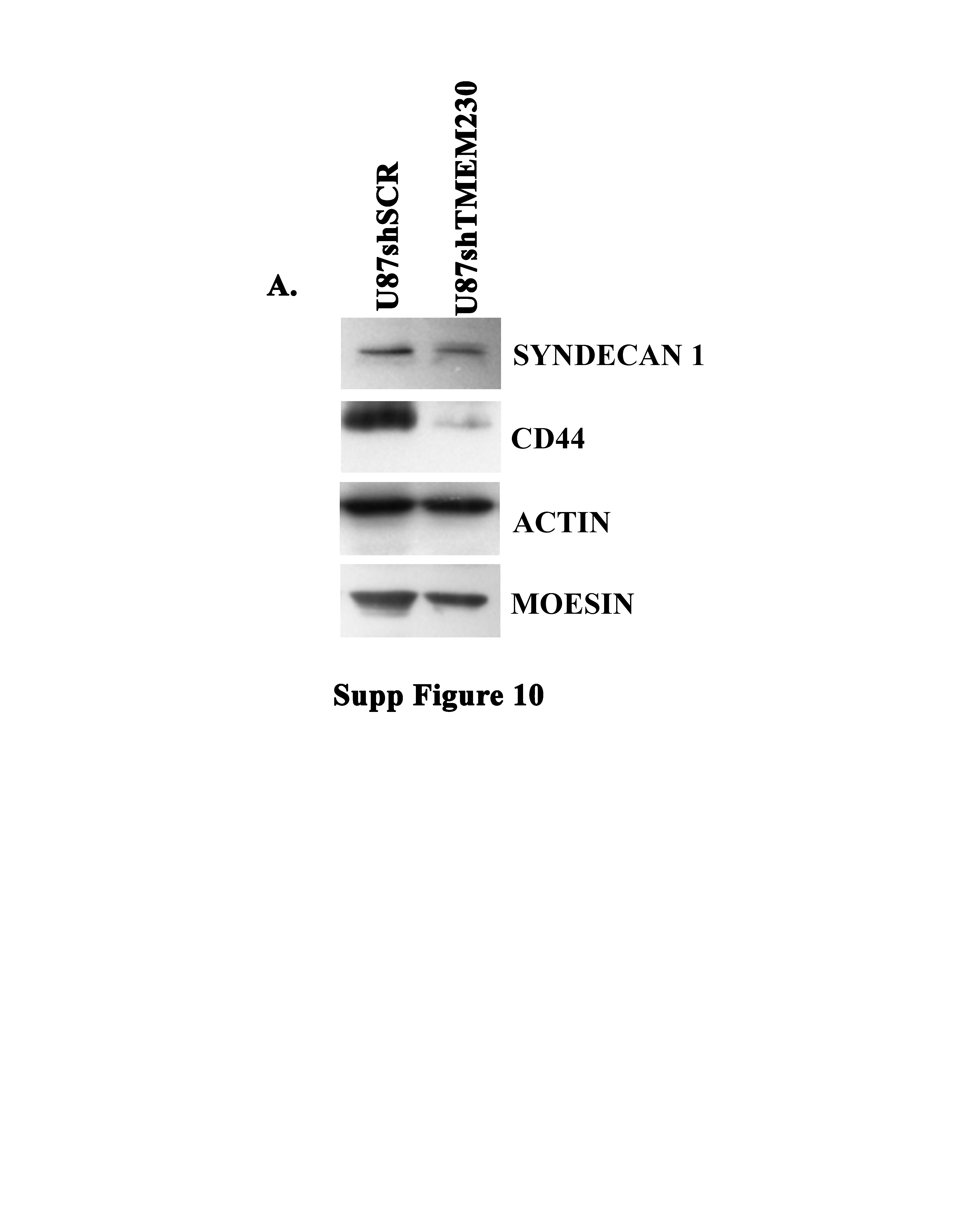

Supplement: Supplementary Figure 10 — Protein validation of TMEM230 candidate responding genes in U87-MG. (A) Western blot analysis for SYNDECAN-1, CD44, and MOESIN proteins in U87 control cells (U87shSCR) and in U87 cells in which TMEM230 was down regulated (U87shTMEM230). Endogenous control: β-ACTIN. (B) Immunofluorescence analysis for CAVEOLIN-1 and ACTIN through phalloidin interaction in U87 control and shTMEM230 transduced cells (GFP staining). Nuclei are visualized with DAPI. That TMEM230 may have a role in actin polymerization and cytoskeleton regulation is supported by immunofluorescence staining of PHALLOIDIN showing that U87 cells in which TMEM230 was downregulated was associated with decrease in the number and quality in the long multistrand structures of actin. (C) Immunofluorescence analysis for FIBRONECTIN in U87 control and shTMEM230 transduced cells (GFP staining). Nuclei are visualized with DAPI. Expression analysis of patient samples suggests that Fibronectin is differentially expressed between gliomas and non-malignant glial cells. Immunofluorescence analysis supports that FIBRONECTIN is not differentially expressed in U87 control and shTMEM230 transduced cells, and therefore TMEM230 does not regulate FIBRONECTIN expression. [file Image_10.PNG]

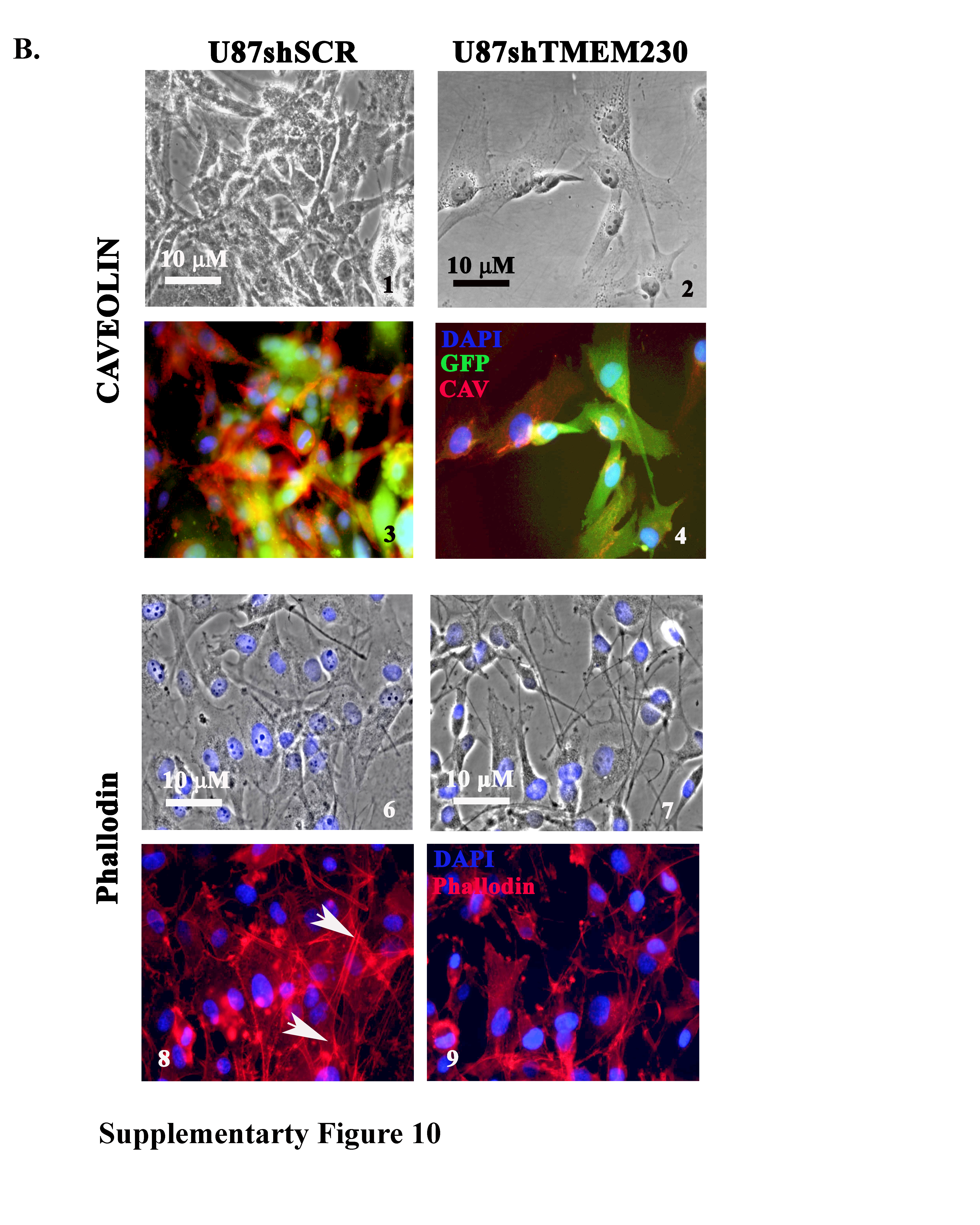

Supplement: Supplementary Figure 11 — TMEM230 expression in diverse cell types from non-malignant and malignant human brain. (A) Distribution of TMEM230 transcripts in different non-malignant cell populations from human brain according to the study of Darmanis (2015) (Accession: GSE67835, survey of human brain transcriptome diversity at the single cell level). Candidate cell populations were identified representative of the major neuronal, glial and vascular cell types according to published candidate gene markers. The distribution is represented by a boxplot with a minimum, the first quartile, the sample median, the third quartile and the maximum of the expression for each cell population, from the bottom to the top. The distribution profiles support that TMEM230 is expressed in most non-malignant cells at relatively equal but low levels. (B–D) Expression analysis of Tmem230 from Single-Cell Rna-Seq Analysis of Infiltrating Neoplastic Cells at the Migrating Front of Human Glioblastoma. (B) 2D-tSne representation of all single cells included from the study of Darmanis (2017) with a sample size of n = 3,589. Cell clusters are differentially colored and identified as distinct cell classes. (C) Expression of characteristic cell-type-specific genes overlaid on the 2D-tSne space. (D) Quantification of Tmem230-positive cells of distinct cell classes. [file Image_11.PNG]

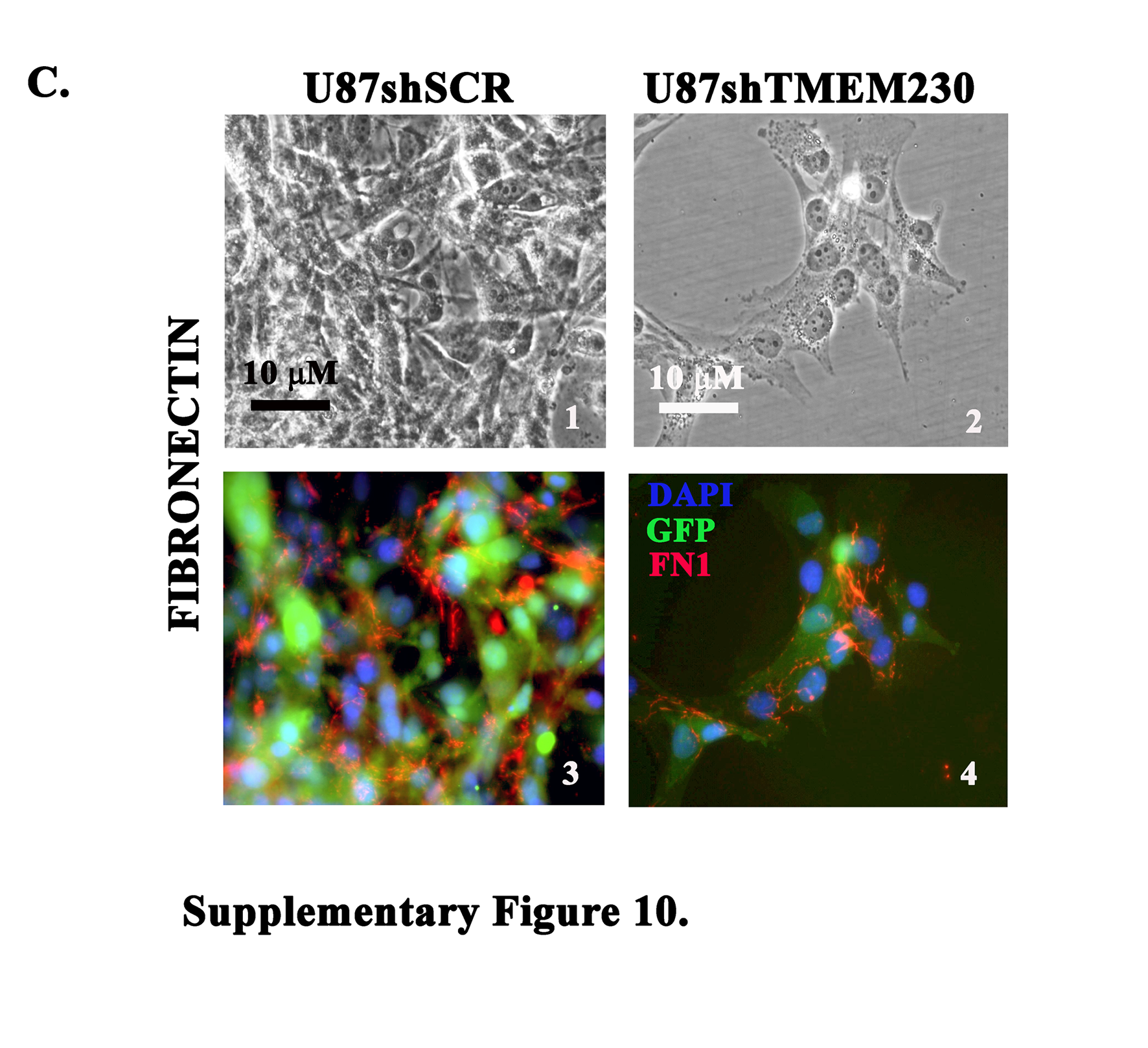

Supplement: Supplementary file 24 [file Image_12.TIF]

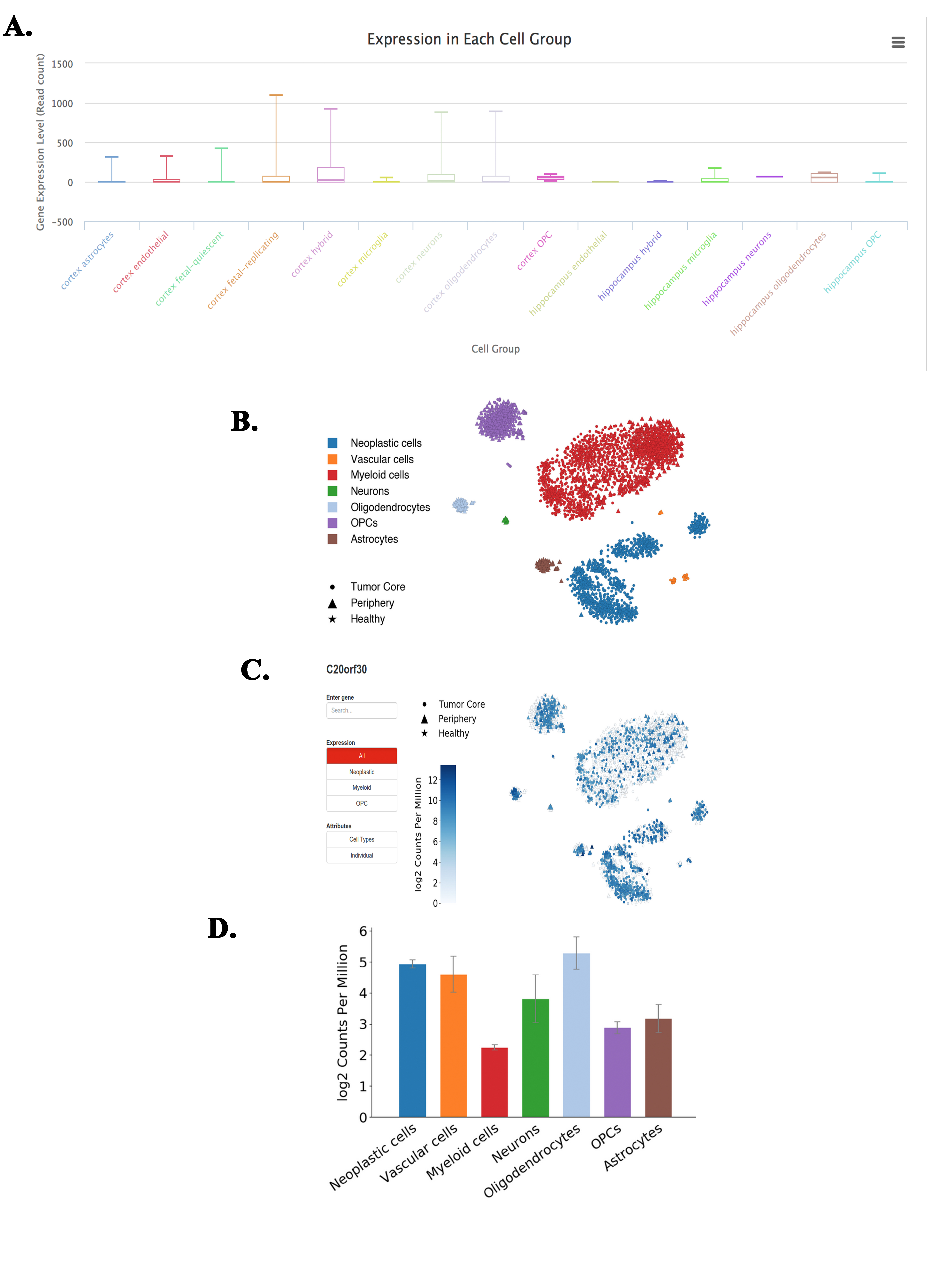

Supplement: Supplementary file 25 [file Image_13.jpg]
